# Enhancing tuber yield and nutraceutical quality of potato by supplementing sunlight with LED red-blue light

**DOI:** 10.3389/fpls.2025.1517074

**Published:** 2025-03-10

**Authors:** Antonio Pannico, Nafiou Arouna, Giovanna Marta Fusco, Piero Santoro, Antonio Giandonato Caporale, Rosalinda Nicastro, Letizia Pagliaro, Stefania De Pascale, Roberta Paradiso

**Affiliations:** ^1^ Department of Agricultural Sciences, University of Naples Federico II, Naples, Italy; ^2^ Department of Environmental, Biological and Pharmaceutical Sciences and Technologies, University of Campania Luigi Vanvitelli, Caserta, Italy; ^3^ Mutable Efficient Growing - MEG S.r.l., Milan, Italy

**Keywords:** *Solanum tuberosum* L., greenhouse, light spectrum, bioregenerative life support systems (BLSSs), GABA, BCAAs, glycoalkaloids

## Abstract

**Introduction:**

We investigated the influence of genetic material and light spectrum on plant performance of two cultivars of potato (*Solanum tuberosum* L.), ‘Colomba’ and ‘Libra’, grown in greenhouse, in the view of future plant cultivation in Space and terrestrial vertical farming and controlled environment agriculture under limiting light conditions.

**Methods:**

The effects of 100% natural light (CNT) and two lighting treatments, in which 30% of solar radiation was replaced by red and blue LED light, RB 1:1 and RB 2:1, were evaluated on plant growth, gas exchange, and tuber yield and quality.

**Results:**

In CNT plants, net photosynthesis (NP) was similar in the cultivars, while the aerial biomass and tuber yield were greater in ‘Libra’. In ‘Colomba’, NP and plant leaf area were unaffected by lighting treatments, however tuber yield increased under RB 2:1. Conversely, in ‘Libra’ both the aerial biomass and tuber production decreased in RB 2:1. Tubers of ‘Colomba’ contained higher concentrations of most minerals than ‘Libra’, probably due to different genetic traits and the slightly lower biomass (concentration effect). Red-blue lighting did not alter the mineral content of tubers. ‘Colomba’ prioritized the accumulation of free amino acids, GABA, and polyphenols, enhancing the plant stress response and antioxidant capacity, and adapted well to variable light conditions, with significant increases in tuber yield under LED treatments. Differently, ‘Libra’ focused on synthesis of carbohydrates, and essential amino acid content was lower compared to ‘Colomba’.

**Discussion:**

Our findings underline the importance of genotype selection and highlights how light spectrum can improve the plant performance in potato. This knowledge could be useful in controlled environment agriculture and indoor cultivation (i.e., vertical farming) as well as in space research on potato, as this crop is a candidate for plant-based regenerative systems for long-term missions.

## 1 Introduction

Fresh food production and bioregeneration have been identified as necessary conditions for future manned interplanetary missions in Space. To accomplish these objectives, intensive studies are carried out to develop Bioregenerative Life Support Systems (BLSSs). BLSSs are artificial ecosystems in which appropriately selected organisms, such as higher plants, algae, and bacteria, are arranged in a series of recycling steps to reconvert the crew waste into oxygen and potable water, while producing foods (Hendrickx and Mergeay, 2007; Lasseur et al., 2010). Higher plants are the most promising bioregenerators because they are able to purify air, recycle water, reuse human waste (like urine and faeces), and provide fresh food and psychological benefits to the astronauts, essential for long-term space missions (Perchonok et al., 2012). However, to carry out efficiently all these functions, plants need to be grown in optimal environmental and cultural conditions.

Light is one of the most important factors in promoting crop productivity as it significantly influences plant growth, development, and metabolisms, and the yield and nutritional quality of products, hence artificial lighting is crucial in cultivation in controlled environment (Ouzounis et al., 2015). Indeed, light provides the energy for photosynthesis and dictates specific signals regulating numerous fundamental processes of plant development, shaping and metabolism, in the complex phenomenon of photomorphogenesis, driven by light colours, perceived by specific plant photoreceptors even at very low light intensity (Devlin et al., 2007). For instance, blue (B), red (R) and far red (FR) promote seedling development and the achievement of autotrophy, implying their ability to perform photosynthesis, and also regulate plant height, branching, leaf expansion, and reproduction; R and B are the most efficient wavelengths in sustaining photosynthesis, and B influences stomatal opening and chlorophyll biosynthesis, and minimizes shade avoidance responses like excessive stem elongation; R-FR ratio can influence seed germination and control flowering in plant species sensitive to photoperiod (Folta and Childers, 2008). Several wavelengths (including B) stimulate the synthesis of antioxidants, which enable plants to react to biotic and abiotic stresses and are healthy compounds for humans (Olle and Viršile, 2013; Thoma et al., 2020).

The combination of R and B has been found more beneficial than the single wavelengths for several vegetable crops (Alrifai et al., 2019; Zheng et al., 2019; Thoma et al., 2020), while only a few data are available for staple crops, like potato, wheat, soybean, typically grown in open field, generally limited to space research (reviewed in Paradiso and De Pascale, 2021). Based on the above-mentioned effects, light quantity (intensity and duration) and quality (spectral composition) have a significant impact on crop productivity and the nutritional and nutraceutical value of vegetables (Hasan et al., 2017). Accordingly, it is conceivable that they influence the plant performance also in staple crops, hence providing artificial lighting at the proper parameters is one of the main challenges not only for growing these plants in BLSSs for Space, but also in all terrestrial environments, characterized by limiting light conditions (such as northern latitudes and prolonged cloudy weather), where solar radiation is usually supplemented with artificial light to improve crop productivity (Tewolde et al., 2018; Palmitessa et al., 2021; Nakayama and Nakazawa, 2023).

In the last decade, modifications of the light intensity, direction, and spectrum in greenhouse environment have been extensively tested in horticulture. These modifications aim at inducing morphological, physiological, and metabolic responses to control plant productivity and to improve product quality (particularly in terms of bioactive compounds). In this respect, artificial lighting with light-emitting diodes (LEDs) (Paradiso and Proietti, 2021) or innovative cover materials with specific optical properties, including photo-selective films (Timmermans et al., 2020; He et al., 2021) and covers doped with light conversion agents modulating the proportion of different wavelengths (Roslan et al., 2018) are studied. LEDs provide many advantages compared to traditional lamps, including the more efficient electrical conversion and the possibility to tune the emitted intensity and spectrum to match the precise light requirements of plant species (Bantis et al., 2018). The effects of light spectrum on plant growth and development have been summarized by several authors (e.g. Fukuda, 2013; Paradiso and Proietti, 2021).

Potato (*Solanum tuberosum* L.) is a candidate species for cultivation in BLSSs, as it is a very productive crop and tubers are a good source of fundamental macromolecules (i.e., starch, proteins, and dietary fiber) (Wheeler, 2006). The high harvest index (0.7-0.8), as a ratio of edible part to total biomass per plant, implies a limited waste to be disposed. Further advantages are the availability of numerous genotypes with different features, the low need for fertilizers, and the staggered harvest of tubers, ensuring a continuous fresh food supply (Wheeler et al., 2019). Potato is traditionally considered a short-day crop, and photoperiod of 12 hours, together with cool temperatures (16-20 °C optimum), are reported to promote tuber production and higher harvest index in several genotypes (Wheeler et al., 1986).

Potato tubers contain glycoalkaloids, which are antinutritional secondary metabolites synthetized in all the plant tissues to protect plants against pathogens and pests, toxic for humans at high concentration (Ginzberg et al., 2009). Acute effects of glycoalkaloids include gastrointestinal disorders and, in severe cases, paralysis, cardiac failure, coma and death, that may occur for a total intake over 1 mg/kg body weight (EFSA Panel on Contaminants in the Food Chain (CONTAM) et al., 2020). The main glycoalkaloids in potato tubers are α-solanine and α-chaconine. The content of glycoalkaloids in potatoes is mainly determined by genetic factors, however, environmental and growing conditions can influence their biosynthesis (Nema et al., 2008). Although there is no maximum level established for food, the total glycoalkaloids (TGA) content in fresh unpeeled tubers below 200 and 250 mg/kg fresh weight is recommended in Europe and USA, respectively (EFSA Panel on Contaminants in the Food Chain (CONTAM) et al., 2020).

Plant physiology of potato has been studied mostly in outdoor conditions, as it is usually cultivated in open field, while only few studies are available on hydroponics under controlled environment. These are mainly space-oriented experiments in growth chamber investigating the influence of different hydroponic systems (i.e., nutrient film technique, potted substrate), agronomical (i.e., nutrient solution recipe) and environmental parameters (i.e., light intensity, photoperiod, air CO_2_ concentration), and alternative or extraterrestrial substrates (i.e., cellulosic sponge, Martian or lunar regolith simulants) carried out by National Aeronautics and Space Administration (NASA; reviewed by Wheeler et al., 2019) and European Space Agency (ESA; Molders et al., 2012; Paradiso et al., 2019, 2020; Caporale et al., 2023), in cultivars selected for BLSSs. However, tuber yield and quality depend on many factors, including genotype, soil characteristics and climatic conditions (Westermann, 2005), and data about the influence of light quality, and particularly of R and B wavelengths, on potato are scarce.

Based on previous evidence, the combination of B and R light could optimize potato tuber production by combining the effects on the earliness and the extension of the tuberization process. However, the best ratio of R and B is not known (Paradiso et al., 2019). The aim of the experiment was to study the effects of two supplemental lighting treatments, with R:B LED light at ratios of 1:1 and 2:1 compared to natural light, on plant growth and tuber yield and nutritional and nutraceutical composition as well as glycoalkaloids content, in two cultivars of potato grown in pot in unheated glasshouse, in winter-spring period. This study will contribute to understand how light spectrum can be modulated to optimize plant productivity and tuber quality, also reducing the energy consumption for artificial lighting in the plant compartment of BLSSs for Space. Besides, this knowledge could have useful terrestrial applications in both vertical farming and controlled-environment agriculture under limiting light conditions.

## 2 Materials and methods

### 2.1 Plant cultivation and experimental treatments

The experiment was carried out at the experimental facilities of the Department of Agricultural Sciences of the University of Naples Federico II (Portici, Italy - 40°49′ N, 14°20′ E), from December 27, 2022 to May 5, 2023. Plants from tuber-seeds of potato (*Solanum tuberosum* L.), pre-sprouted in growth chamber in the dark, at 18 °C and 70% relative humidity, were grown on a peat-based substrate, in plastic pot (21 cm diameter) placed on benches, in an unheated glasshouse.

**Figure 1:**
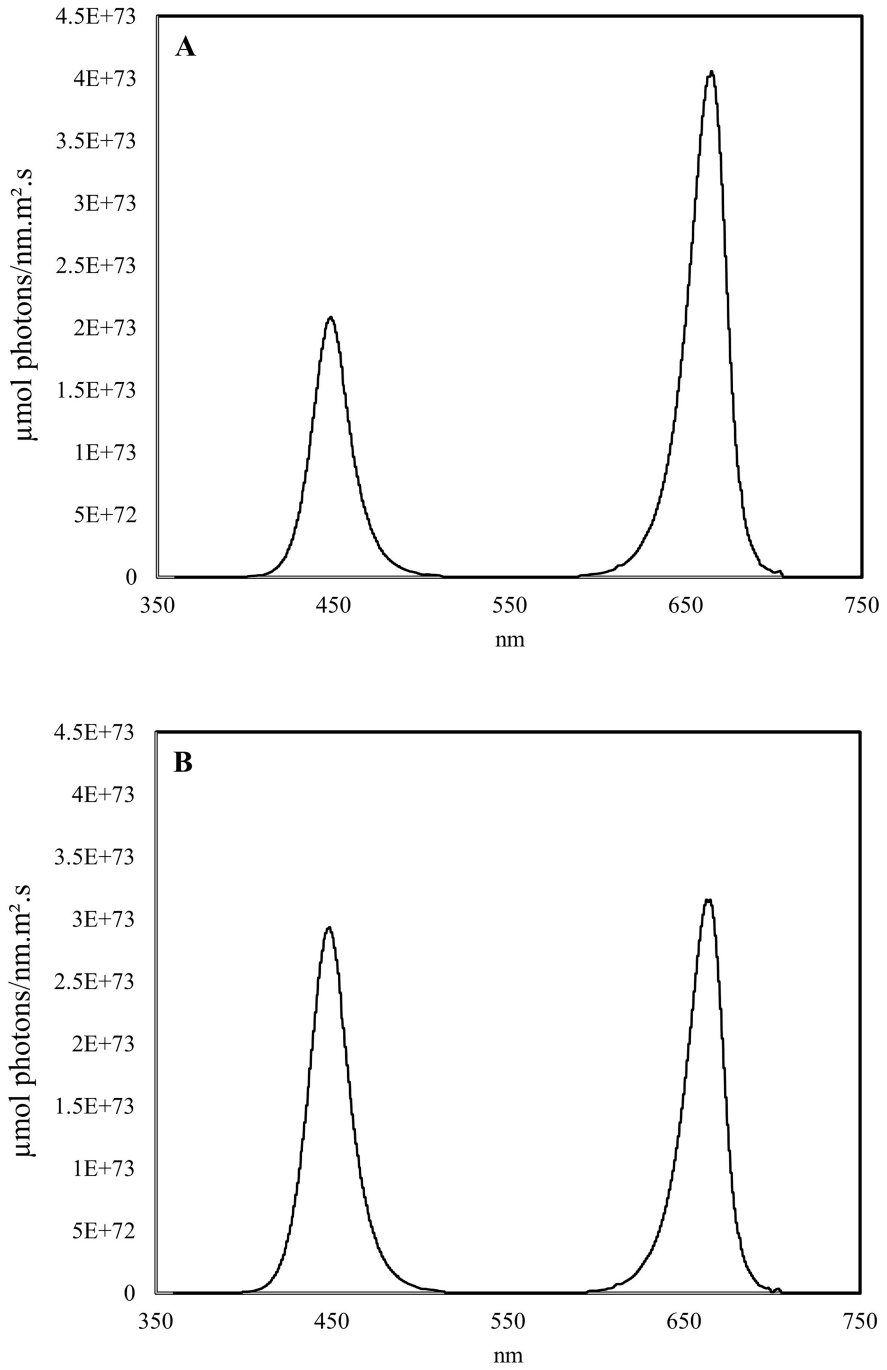
Spectral distribution of the red-blue LED light of supplemental lighting treatments RB 2:1 **(A)** and RB 1:1 **(B)** applied in the experiment on greenhouse cultivation of potato (*Solanum tuberosum* L.).

The influence of lighting treatments was evaluated in two cultivars, ‘Colomba’ and ‘Libra’ (HZPC Holland B.V.), selected as suitable for cultivation in BLSS, based on ESA criteria, including small size, short duration of the growing cycle, and high productivity. Three light treatments were applied: a 100% natural light control (CNT) and two artificial light treatments in which, using a shading net placed over the LED panels, about 30% of solar radiation was replaced by red and blue LED light at the ratios 1:1 and 2:1 (RB 1:1 and RB 2:1, respectively). For this purpose, two plots were equipped with nets at 30% shading to simulate limiting light conditions, and a customized automated system with B (peak 450 nm) and R (peak 660 nm) LEDs was built to provide light supplementation. This system was able to dynamically integrate the solar radiation with artificial light while keeping the Daily Light Integral (DLI) equal to that of the natural light control (unshaded plot). Specifically, a control unit dimmed in real time the radiation emitted by LEDs based on measurements of PAR sensors (LPPAR03, Delta OHM, Padova, Italy) placed at the plant level in the shaded plots and the unshaded control. The normalized spectra of LED arrays are shown in **Figure 1**, while the average DLI throughout the experiment was 12.2 mol m^-2^ d^-1^. The experimental design consisted of randomized blocks with 3 repetitions, each block including 4 plants per cultivar for each light treatment (3 repetitions x 4 plants x 2 cultivars = 24 plants in total per light treatment).

During the experimental period, the natural day length increased from 9 hours and 25 minutes on December 28, 2022 (1 day after sowing - DAS) to 14 hours and 7 minutes on May 6, 2023 (130 DAS). In the control plot under natural light, the DLI varied from 1.8 mol m^-2^ d^-1^ on January 16 (20 DAS) to 26.2 mol m^-2^ d^-1^ on May 6 (130 DAS). Air temperature and relative humidity were recorded every 15 minutes using a WatchDog A150 data logger (Spectrum Technologies Inc., Aurora, IL, USA), during the entire experiment. The average day temperature ranged between 12.3 ± 3.5 °C (2^nd^ decade of January) to 29.2 ± 2.6 °C (3^rd^ decade of April), while the average night temperature fluctuated from 3.36 ± 2.61 °C (first decade of February) to 18.0 ± 0.7 °C (first of May) (Mean Value ± Standard Deviation). The experiment was carried out at ambient CO_2_ concentration (about 435 ppm on average).

Fertigation was performed using a complete nutrient solution based on the formula described by Molders et al. (2012), with pH 5.5 and EC 1.8 dS m^-1^. Plants were fertigated manually three times per week, until container capacity. Every three pulses, fertigation was alternated with one irrigation with deionized water to prevent the salt accumulation in the substrate.

### 2.2 Gas exchange, chlorophyll *a* fluorescence and leaf greenness

Physiological measurements were carried out from 11:00 to 13:00, in different times of the growing cycle, corresponding to different stages of plant development: chlorophyll fluorescence and SPAD index at 79 DAS and 101 DAS (vegetative growth), and at 129 DAS (fully developed plants, just before the harvest), and gas exchange at 101 DAS and 129 DAS. The leaf gas exchange, in terms of net CO_2_ assimilation rate (NP), stomatal conductance (gs), and transpiration rate (E), was measured using a portable infra-red gas-analyser LCi T (ADC BioScientific Ltd., Hoddesdon, United Kingdom), equipped with a clear broad-leaf plant leaf chamber (cuvette area 6.25 cm^2^). Measurements were carried out on the middle leaflet in one fully expanded and well-exposed leaf in the middle part of the main stem, in 6 plants per treatment (2 plants per replicate). The conditions inside the chamber were kept as follows: ambient light (according to light treatments), ambient temperature, ambient CO_2_ concentration, and air flow rate 400 ml min^-1^. During gas exchange measurements in the light, the Photosynthetic Photon Flux Density (PPFD) at canopy level was on average 373 ± 37 and 524 ± 66 µmol m^-2^ s^-1^ at 101 and 129 DAS, respectively (Mean value ± Standard deviation).

On the same leaves, chlorophyll *a* fluorescence emission was measured with a portable fluorometer (Plant stress Kit, Opti-Sciences, Hudson, NY, USA), on 6 plants per treatment, at ambient temperature. The basal signal of fluorescence (F_o_) was induced on 30 min dark adapted leaves, by a blue LED internal light of about 1-2 µmol photons m^-2^ s^-1^. The maximal fluorescence in the dark (F_m_) was triggered by 1 second saturating light pulse of 3000 µmol photons m^-2^ s^-1^. The maximum quantum efficiency of PSII (F_v_/F_m_) was calculated as (F_m_-F_o_)/F_m_ according to Kitajima and Butler (1975). The measurements in the light were conducted at the ambient PPFD. The quantum yield of PSII electron transport (QY) was determined by means of an open leaf-clip, according to Genty et al. (1989). QY was used to calculate the linear electron transport rate (ETR), according to the equation of Krall and Edwards (1992).

The leaf greenness was measured with a portable SPAD-502 chlorophyll meter (Konica Minolta Co., Osaka, Japan) and expressed as SPAD (Soil Plant Analysis Development) units. Measurements were carried out on 10 leaves per plant in 6 plants (2 plants per repetition, 60 measurements per treatment in total), immediately prior to the harvest.

### 2.3 Plant growth and tuber yield

Plant height, number of leaves and plant leaf area were recorded in 3 plants per replicate (6 plants per treatment) at the end of the experiment (129 DAS). Leaf area was estimated by digital image analysis of all the leaves per plant with the ImageJ v1.52a software (Wayne Rasband National Institute of Health, Bethesda, MD, USA).

At the harvest, the fresh weight of leaves, stems, roots and tubers, and the dry biomass of each plant portion after oven drying at 80 °C until constant weight were determined with an analytic balance (BL 2002 basic, XS Instruments, Italy). Tuber yield was determined as number of tubers and total weight (g per plant). The harvest index (HI) was calculated as a ratio of edible part to total biomass per plant.

### 2.4 Leaf chlorophylls and carotenoids

The determination of chlorophyll and carotenoid content followed the methodology of Wellburn (1994) with slight adjustments. A 10 mg sample of frozen leaf powder was mixed with 1 mL of methanol and centrifuged at 13,500 g for 10 minutes. Clear supernatant (100 µL) was transferred into a polypropylene microplate for the absorbance measurement using a Synergy HT microplate reader (BioTEK Instruments, Bad Friedrichshall, Germany). Chlorophyll a (Chl a), chlorophyll b (Chl b), and total carotenoids were estimated at 665 nm, 652 nm, and 470 nm, respectively, and the concentrations were calculated in µg g^−1^ DW.

### 2.5 Multielement profile of potato tubers

The total concentration of the main minerals (K, P, Mg, Ca, Na, Fe, Zn, Mn, B, Cu) in the tubers was determined in acid-digested samples by Inductively Coupled Plasma - Mass Spectrometry (ICP-MS, Thermo Scientific iCAP Q, Waltham, MA, USA). Blanks and standards of known element concentrations were used for instrument calibration and ongoing analytical checks. The standard reference material NCS ZC85006 was used to monitor the quality of analyses, with element recoveries around ±10% of the certified values. The digestion of freeze-dried and pulverized tuber samples (100 mg each) was performed in a microwave digestion system (Milestone UltraWave, Sorisole, BG, Italy), through a blend of HNO_3_ 65% (2.5 ml) and 3M HCl 37% (0.5 ml). The acid extracts were adequately diluted with ultrapure water before the analysis with ICP-MS. The choice of the isotopes to analyse for each element was based on the non-isobaric overlap from other elements in the sample, and the abundance and absence of polyatomic ion interferences formed from precursors in the plasma gas, water, acids used for sample digestion and extract dilutions. The following isotopes were chosen: 11B, 44Ca, 65Cu, 56Fe, 39K, 31P, 24Mg, 23Na, 55Mn and 66Zn. Additionally, element concentrations were acquired in collision mode, using a non-reactive gas (i.e., helium) and a process of kinetic energy discrimination (KED) to selectively attenuate all polyatomic interferences based on their size.

### 2.6 Metabolic profile of potato leaves and tubers

#### 2.6.1 Starch and soluble carbohydrate

A 10 mg sample of frozen material powder was extracted twice with 150 mL of 80% ethanol (v:v), and then re-extracted using 150 mL of 50% ethanol (v:v) at 80 °C for 30 minutes. After centrifugation at 13,500 g for 10 minutes at 4 °C, the clear supernatant from all extractions was pooled and stored at -20 °C for subsequent sugar analysis. The remaining pellet was treated with 350 mL of 0.1 M KOH and heated at 95 °C for 2 hours for starch hydrolysis. Once cooled, the samples were acidified to pH 4.5 and combined 1:1 with a hydrolysis buffer containing 50 mM sodium acetate (pH 4.5), 2 U/mL α-amylase, and 20 U/mL amyloglucosidase, and incubated at 37 °C for 18 hours. The resulting mixture was centrifuged at 13,500 g for 10 minutes at 4 °C, and the supernatant, containing glucose from the hydrolysed starch, was collected for measurement. Glucose, fructose, and sucrose content in the ethanol extracts and starch-derived glucose were quantified using an enzymatic assay coupled with pyridine nucleotide reduction as described by Carillo et al. (2019) and expressed in µmol g^-1^ DW.

#### 2.6.2 Soluble proteins and free amino acid

The soluble protein content was assessed following a modified version of the Bradford, (1976). A 10 mg sample of freeze-dried material was extracted using a buffer with 200 mM TRIS-HCl (pH 7.5) and 500 mM MgCl_2_ at 4 °C for 24 hours. After centrifuging at 13,500 g for 5 minutes at 4 °C, a 20 µL aliquot of the supernatant was mixed with 180 µL of diluted protein assay reagent (Bio-Rad, Milan, Italy) in a 1:5 ratio with Milli-Q water. Protein concentration was determined against standard curves of bovine serum albumin (BSA) and expressed in mg g^-1^ DW. For amino acid analysis, 10 mg of leaves or tubers was extracted in 1 mL of ethanol (40:60 v:v) overnight at 4 °C. Quantification was performed by HPLC (Nexera LC-30, Shimadzu Europa GmbH, Duisburg, F.R., Germany) following pre-column derivatization with *o*-phthaldialdehyde (OPA) as described by Dell’Aversana et al. (2021). Proline content was determined using the same extract according to Carillo et al. (2019). Results were expressed in µmol g*^−^* ^1^ DW.

#### 2.6.3 Polyphenols

Aliquots of 10 mg of frozen material powder were suspended in 700 µL of 60% methanol (v:v), and centrifuged at 13,500 *g* for 10 min at 25 °C. Clear supernatant aliquots (35 µL) were combined with 125 µL of diluted Folin–Ciocalteu reagent (1:1 with Milli-Q water) and 650 µL of 3% sodium carbonate solution w:v) sodium carbonate. After a 90-minute reaction at room temperature, absorbance was measured at 760 nm using a Synergy HT spectrophotometer (BioTEK Instruments, Bad Friedrichshall, Germany), following the method described by Rouphael et al. (2020). Total phenol content was calculated using a standard curve with known concentrations of gallic acid (GAE) and expressed as mg GAE equivalents g*^−^* ^1^ DW.

#### 2.6.4 Glycoalkaloids

Glycoalkaloid analysis was performed following a modified procedure from Singh et al. (2016). A 0.5 g aliquot of frozen tubers powder was extracted twice with 7 mL of a solution of water, acetic acid, and sodium bisulfate (95:5:0.5, v:v:v), at room temperature for 30 minutes. After centrifuging at 13,500 g for 10 minutes, the clear supernatants were pooled, and 3 mL were filtered through a Clarify-NY 13 mm-0.45 µm syringe filter, pre-conditioned sequentially with 5 mL acetonitrile and the extraction solvent. The filter was then rinsed with 5 mL of a water and acetonitrile (85:15, v:v) solution, and glycoalkaloids were eluted using 3 mL of acetonitrile and 0.022 M phosphate buffer pH 7.6 (55:45, v:v). Glycoalkaloids were measured using an HPLC system with a reverse-phase RP-18 column and a mobile phase of acetonitrile, 0.022 M phosphate buffer and tetrahydrofuran (55:44:1, v:v:v) at a flow rate of 1.5 mL/min and an injection volume of 20 μL. The retention times were around 3 minutes for α-solanine and 4 minutes for α-chaconine, detected at 208 nm using a diode-array spectrophotometer (HP 8452A, Agilent Technologies, Palo Alto, CA, USA), quantified by comparison with standard solutions under identical conditions, and expressed as mg kg*^−^* ^1^ DW.

### 2.7 Statistical analysis

Six treatments, derived by the factorial combination of two cultivars (C) and three light conditions (L), were compared. Cultivars were arranged under the lighting treatments in a randomized block design with three replicates. All experimental data were analyzed by two-way analysis of variance (ANOVA), using the SPSS 29 software package (IBM, Armonk, NY, USA). To compare the means of each measured parameter, a Tukey *post hoc* test was performed at the significance levels of p ≤ 0.05, p ≤ 0.01, and p<0.001.

## 3 Results

### 3.1 Plant growth and physiology

**Figure 2:**
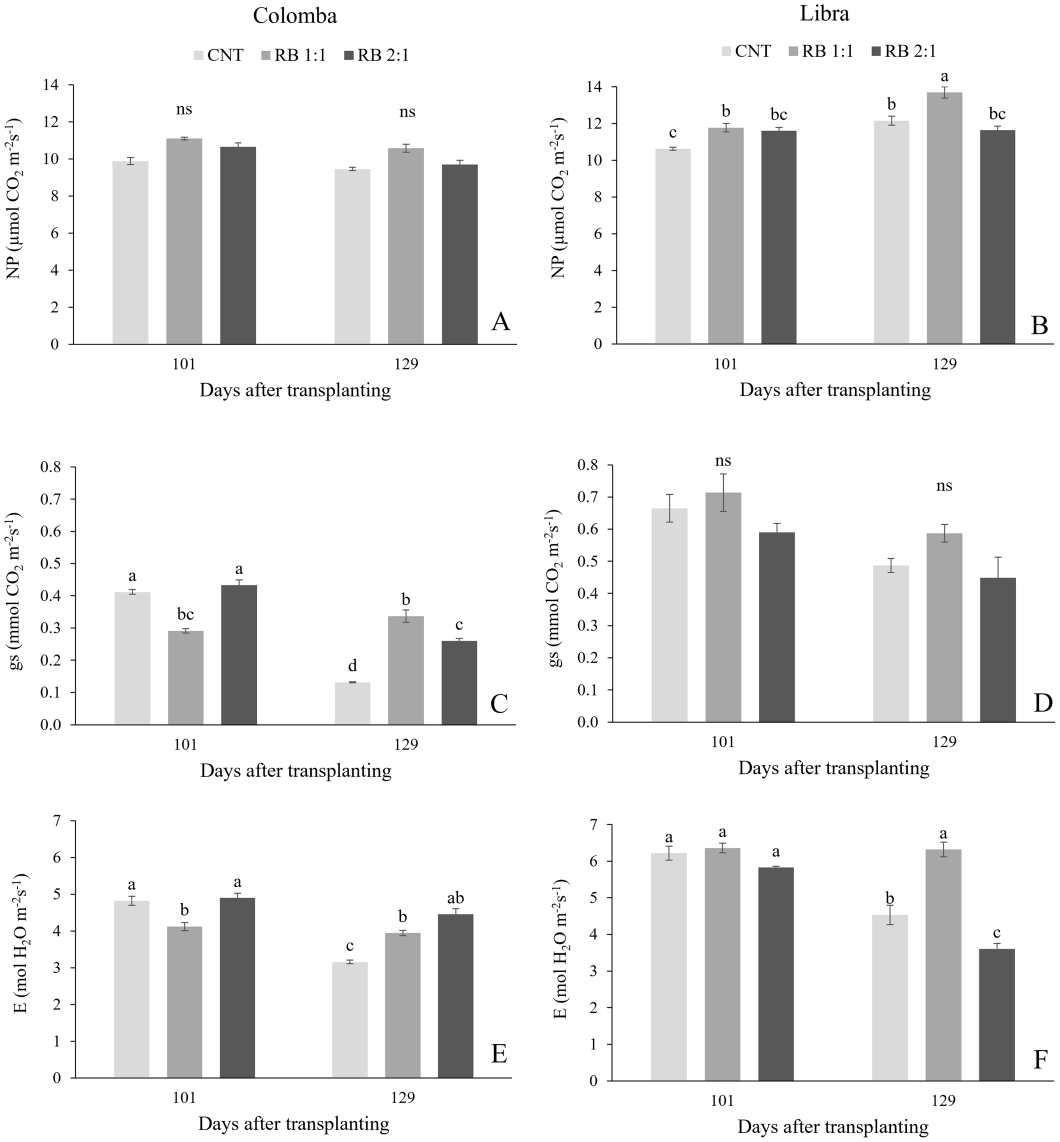
Net-photosynthesis (NP), stomatal conductance (gs) and transpiration rate (E) measured in potato plants cv. 'Colomba' **(A, C, E)** and 'Libra' **(B, D, F)** grown under natural light (control, CNT) and natural light shaded at 30% and integrated with red and blue LED light at 1:1 (RB 1:1) and 2:1 (RB 2:1) ratios. Mean value ± Standard error, n=6. Different letters indicate significant differences, ns, not significant differences among the treatments according to Tukey's multiple-range test at p<0.05.

**Figure 3:**
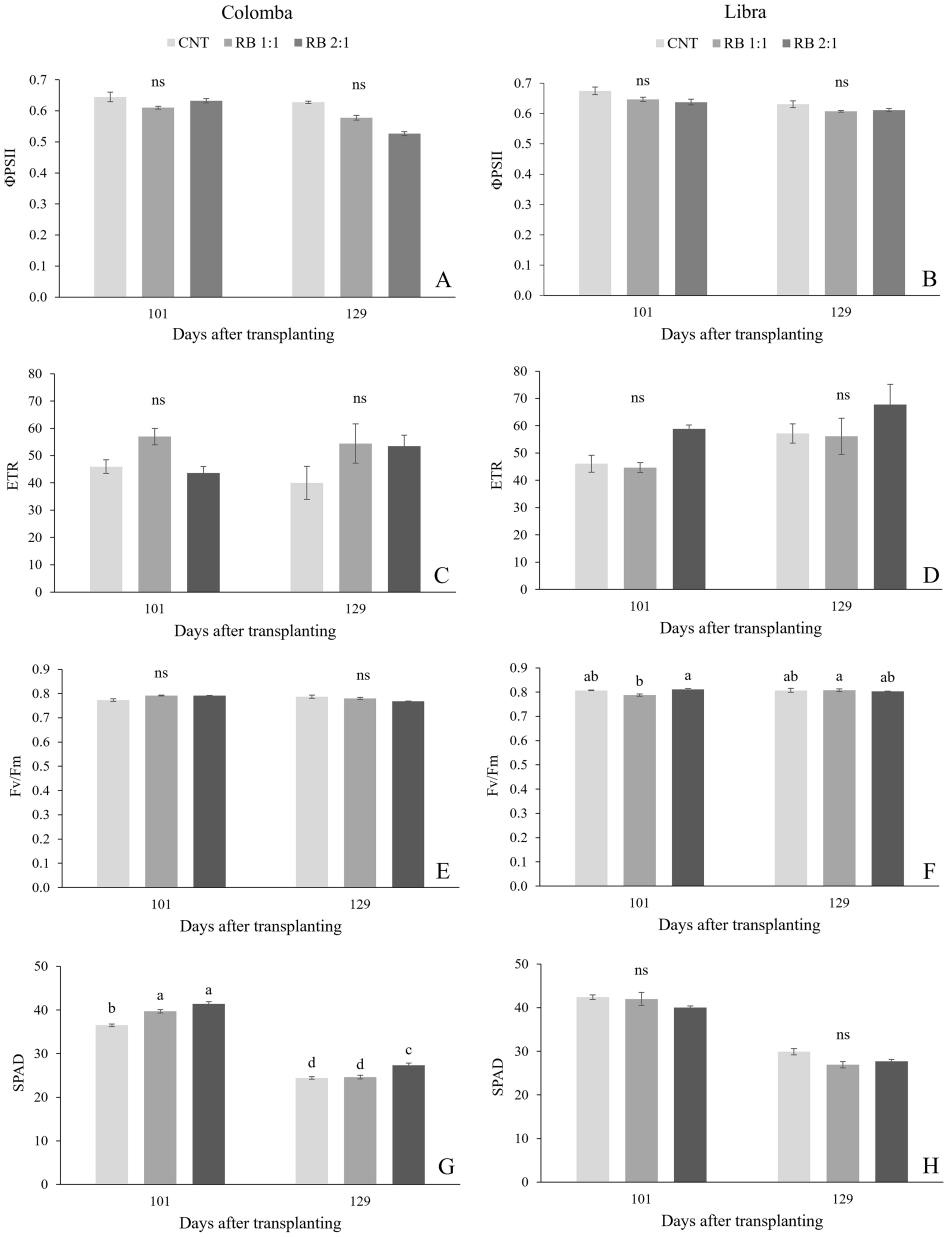
The PSII quantum yield of linear electron transport (ΦPSII), electron transport rate (ETR), maximum quantum efficiency of PSII photochemistry (Fv/Fm) and leaf greenness, expressed as SPAD index measured in potato plants cv. 'Colomba' **(A, C, E, G)** and 'Libra' **(B, D, F, H)** grown under natural light (control, CNT) and natural light shaded at 30% and integrated with red and blue LED light at 1:1 (RB 1:1) and 2:1 (RB 2:1) ratios. Mean value ± Standard error, n=6. Different letters indicate significant differences, ns not significant differences among the treatments according to Tukey's multiple-range test at p<0.05.

Regarding the effect of cultivar, measurements of foliar gas exchanges showed significantly higher values of net photosynthesis (NP), stomatal conductance (gs) and transpiration (E) in ‘Libra’ than in ‘ Colomba’ (**Supplementary Table S1**; **Figure 2**). Similarly, the quantum yield of PSII electron transport (ΦPSII), the maximal PSII photochemical efficiency (F_v_/F_m_) and leaf greenness, expressed as SPAD index, were significantly higher in ‘Libra’ than in ‘ Colomba’ (**Supplementary Table S1** and **Figure 3**).

In plants of cv. ‘Colomba’, NP did not change in the different lighting treatments (**Figure 2A**), whereas gs was significantly lower under RB1:1 compared to natural light conditions (CNT) at 101 DAS; however, gs remained constant under RB 1:1, resulting in the highest value at 129 DAS, since it decreased in the other treatments (**Figure 2C**). Similarly, at 101 DAS, E was significantly lower in RB1:1 than in CNT, while at 129 DAS it was significantly higher in both supplemental light treatments than in CNT (**Figure 2E**). In ‘Libra’, RB 1:1 light promoted NP significantly at both the dates, with the highest value reached during the tuber bulking (**Figure 2B**). In contrast, no relevant difference was observed in gs in the different lighting treatments and dates (**Figure 2D**), while only at 129 DAS, E in RB1:1 was significantly higher than in the two light treatments (**Figure 2F**).

**Figure 3** shows the photochemical parameters recorded in the same leaves sampled for gas exchange measurements, at the same dates. Under CNT, the ΦPSII and the linear electron transport rate (ETR) were similar in the cultivars and did not change over time. In both the cultivars, lighting treatments did not influence photochemical parameters at both the dates (**Figures 3A-F**). In plants of ‘Colomba’, SPAD values greatly increased under RB 1:1 and RB 2:1 compared to CNT in vegetative growth, then decreased during the bulking stage (129 DAS) (**Figure 3G**), while in ‘Libra’ the leaf greenness was unaffected by lighting conditions (**Figure 3H**).

The two cultivars showed different responses to lighting treatments in terms of growth of both the aerial and the hypogeous parts (**Tables 1**, **2**).

**Table 1:** Main growth parameters in the aerial part of potato plants cv. ‘Colomba’ and ‘Libra’ grown under natural light (control, CNT) and natural light shaded at 30% and integrated with red and blue LED light at 1:1 (RB 1:1) and 2:1 (RB 2:1) ratios.

Source of variance		No. of leaves	Plant leaf area	Specific leaf area	Leaves DW	Total aerial biomass
		(no. plant^-^¹)	(cm^2^ plant^-1^)	(cm^2^ leaf^-1^)	(g plant^-1^)	(g D.M. plant^-1^)
Cultivar (C)	Colomba	10.58 ± 0.66 b	233.59 ± 5.80 b	22.65 ± 1.25 b	1.08 ± 0.04 b	1.17 ± 0.04 b
	Libra	13.65 ± 0.34 a	402.92 ± 14.03 a	29.49 ± 0.58 a	2.14 ± 0.07 a	2.57 ± 0.08 a
Light treatment (L)	CNT	11.46 ± 1.46	336.81 ± 50.38	29.00 ± 0.97 a	1.62 ± 0.3 ab	1.92 ± 0.39 a
	RB 1:1	12.22 ± 0.56	313.91 ± 37.57	25.38 ± 2.20 b	1.71 ± 0.24 a	1.96 ± 0.31 a
	RB 2:1	12.67 ± 0.36	304.03 ± 28.14	23.83 ± 1.73 b	1.49 ± 0.18 b	1.73 ± 0.24 b
C x L						
Colomba	CNT	8.30 ± 0.66 c	225.99 ± 10.88 c	27.37 ± 0.83 a	0.96 ± 0.06 c	1.05 ± 0.05 d
	RB 1:1	11.33 ± 0.58 b	232.78 ± 10.68 c	20.62 ± 1.16 b	1.18 ± 0.04 c	1.27 ± 0.03 c
	RB 2:1	12.12 ± 0.59 ab	241.98 ± 10.34 c	19.97 ± 0.17 b	1.09 ± 0.01 c	1.19 ± 0.02 cd
Libra	CNT	14.63 ± 0.47 a	447.63 ± 17.01 a	30.64 ± 1.15 a	2.28 ± 0.04 a	2.79 ± 0.03 a
	RB 1:1	13.11 ± 0.68 ab	395.05 ± 18.96 ab	30.14 ± 0.26 a	2.23 ± 0.08 a	2.65 ± 0.08 a
	RB 2:1	13.22 ± 0.11 ab	366.07 ± 1.64 b	27.69 ± 0.32 a	1.89 ± 0.00 b	2.27 ± 0.02 b
Significance						
Cultivar (C)		***	***	***	***	***
Light treatment (L)		ns	ns	***	**	***
C x L		***	**	**	***	***

Mean values ± Standard Errors; n=3. Different letters indicate significant differences according to Tukey’s multiple-range test (p<0.05). ns, not significant; ** p<0.01; *** p<0.001.

**Table 2:** Main growth parameters in the hypogeous part of in potato plants cv. ‘Colomba’ and ‘Libra’ grown under natural light (control, CNT) and natural light shaded at 30% and integrated with red and blue LED light at 1:1 (RB 1:1) and 2:1 (RB 2:1) ratios.

Source of variance		Number of tubers (N./plant)	Tuber width (mm)	Tuber length (mm)	Tuber yield (g plant^-1^)	Specific tuber FW (g tuber^-1^)	Tubers DW (g plant^-1^)	Root DW (g plant^-1^)	Hypogeous biomass (g D.M. plant^-1^)	Tuber D.M (% of fresh weight)
Cultivar (C)	Colomba	3.88 ± 0.15 a	35.91 ± 0.43 b	40.43 ± 0.85 b	119.53 ± 3.43 b	30.93 ± 0.63 b	16.94 ± 0.54 b	1.00 ± 0.06 b	17.95 ± 0.54 b	14.17 ± 0.12 b
	Libra	3.37 ± 0.14 b	38.35 ± 0.52 a	45.08 ± 0.97 a	130.95 ± 3.73 a	39.19 ± 1.25 a	21.44 ± 0.52 a	3.26 ± 0.15 a	24.69 ± 0.56 a	16.43 ± 0.40 a
Light treatment (L)	CNT	3.53 ± 0.09	36.68 ± 0.76	42.2 ± 1.65	124.53 ± 6.65	35.33 ± 1.98	18.11 ± 1.21 b	2.32 ± 0.58	20.43 ± 1.78 b	14.49 ± 0.24 b
	RB 1:1	3.68 ± 0.13	37.73 ± 0.67	43.7 ± 0.94	126.56 ± 4.74	34.69 ± 2.20	19.79 ± 1.54 a	2.16 ± 0.55	21.95 ± 2.07 a	15.52 ± 0.64 a
	RB 2:1	3.66 ± 0.33	36.97 ± 0.91	42.36 ± 1.83	124.63 ± 3.73	35.15 ± 2.47	19.67 ± 0.43 a	1.91 ± 0.41	21.58 ± 0.76 a	15.88 ± 0.72 a
C x L										
Colomba	CNT	3.51 ± 0.15 bc	35.37 ± 0.39	39.89 ± 1.94	109.91 ± 0.99 b	31.49 ± 1.54	15.44 ± 0.29 c	1.04 ± 0.05	16.48 ± 0.31 d	14.05 ± 0.20 b
	RB 1:1	3.78 ± 0.11 ab	37.05 ± 0.75	42.36 ± 0.73	116.13 ± 0.27 b	30.79 ± 0.81	16.42 ± 0.35 c	0.95 ± 0.03	17.37 ± 0.34 d	14.14 ± 0.27 b
	RB 2:1	4.36 ± 0.17 a	35.29 ± 0.72	39.03 ± 1.17	132.56 ± 1.78 a	30.52 ± 1.21	18.97 ± 0.03 b	1.01 ± 0.18	19.98 ± 0.18 c	14.32 ± 0.18 b
Libra	CNT	3.56 ± 0.11 abc	38.00 ± 1.01	44.51 ± 2.13	139.16 ± 2.53 a	39.18 ± 1.54	20.78 ± 0.41 b	3.61 ± 0.17	24.38 ± 0.41 b	14.93 ± 0.20 b
	RB 1:1	3.59 ± 0.26 abc	38.41 ± 1.12	45.05 ± 1.44	136.98 ± 1.87 a	38.59 ± 2.90	23.16 ± 0.57 a	3.36 ± 0.22	26.52 ± 0.57 a	16.90 ± 0.20 a
	RB 2:1	2.96 ± 0.16 c	38.65 ± 0.91	45.69 ± 2.09	116.71 ± 1.92 b	39.78 ± 2.76	20.37 ± 0.66 b	2.8 ± 0.16	23.17 ± 0.58 b	17.45 ± 0.30 a
Significance										
Cultivar (C)		**	**	**	***	***	***	***	***	***
Light treatment (L)		ns	ns	ns	ns	ns	**	ns	**	***
C x L		**	ns	ns	***	ns	***	ns	***	***

Mean values ± Standard Errors; n=3. Different letters indicate significant differences according to Tukey’s multiple-range test (p<0.05). ns, not significant; ** p<0.01; *** p<0.001.

The analysis of the main effects (**Table 1**) showed that all the growth parameters in the aboveground part were significantly influenced by both genotype and light quality, except the number of leaves and plant leaf area, on which lighting treatments had no significant impact. Averaged on the lighting treatments, all the growth parameters were significantly higher in ‘Libra’ than in ‘Colomba’. However, significant interactions were found between cultivars and lighting conditions for all the measured parameters. In ‘Colomba’, the number of leaves significantly increased under RB 1:1 (+36.5%) and RB 2:1 (+46%) compared to CNT (**Table 1**). However, in ‘Colomba’ no significant difference was found among the treatments in plant leaf area, since RB light enrichment reduced the specific leaf area, and the total aerial biomass increased only under RB 1:1 (+21%) compared to CNT. Regarding ‘Libra’, plant leaf area decreased under RB 2:1 (-22.3% than CNT), resulting in significantly lower values of total aerial biomass (-18.6% compared to CNT).

The analysis of the main effects (**Table 2**) revealed that all underground growth parameters were significantly influenced by the genotype, with the higher values recorded in ‘Libra’ compared to ‘Colomba’, except that the number of tubers per plant. Averaged on the cultivars, the light quality had a significant impact only on tubers DW, total hypogeous biomass and tuber DM, with highest averages recorded in RB 1:1 and RB 2:1 compared to CNT. In ‘Colomba’, lighting treatments did not influence the size, FW and dry matter (DM) of tubers and the root DW (**Table 2**). In contrast, the number of tubers and the yield per plant increased under RB 2:1, with consequent increase in tuber DW and total hypogeous biomass compared to CNT. As for ‘Colomba’, light did not influence the tuber dimensions and FW, the DW of tubers and roots and the overall hypogeous biomass in ‘Libra’; however, a different trend was observed in tuber yield, which was reduced by RB 2:1 light, and tuber DM percentage, which increased under both the RB treatments compared to CNT (**Table 2**).

**Figure 4:**
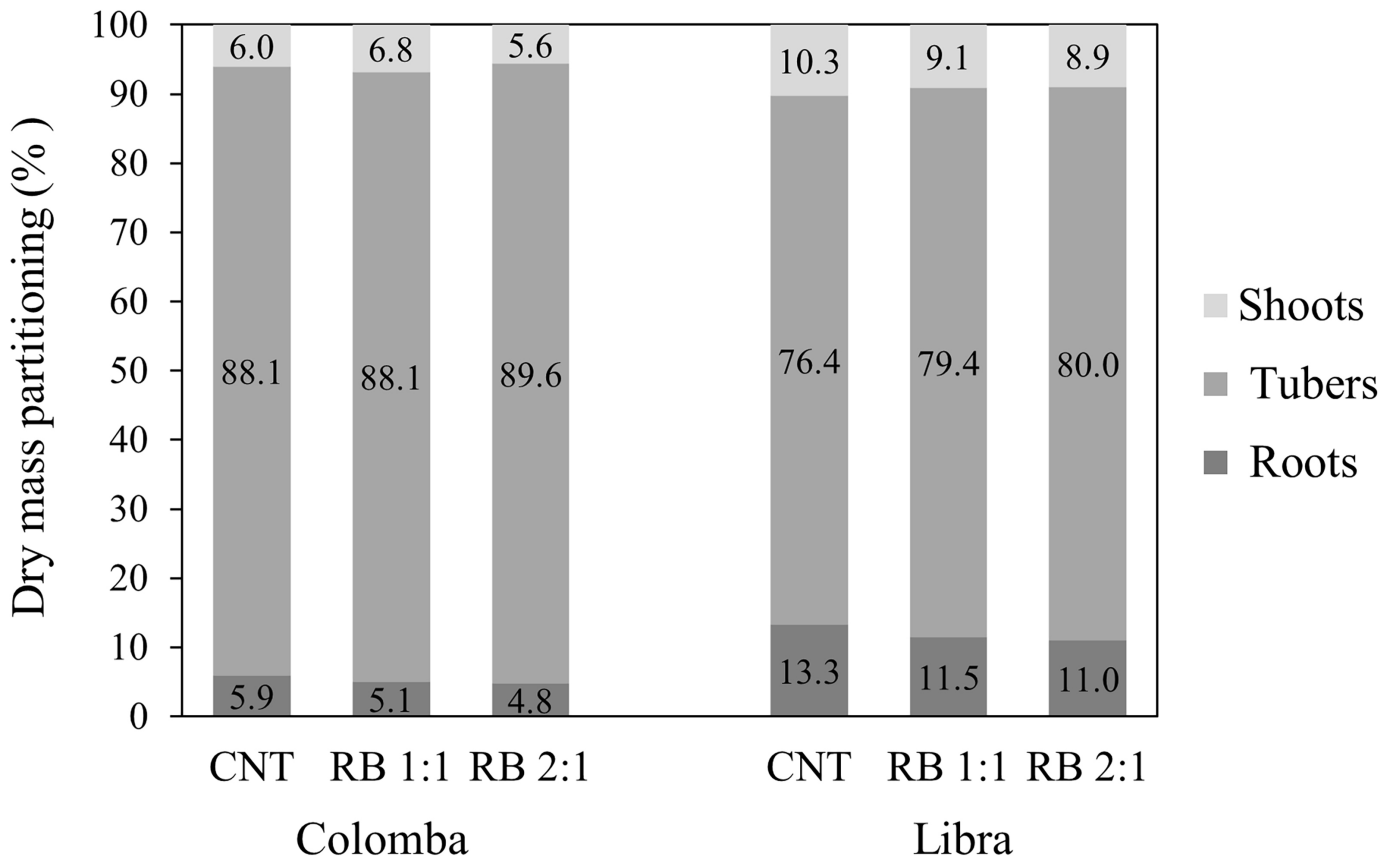
Total dry matter partitioning in the plant organs (percentage of total dry biomass) in potato cv. ‘Colomba’ and ‘Libra’ grown under natural light (control, CNT) and natural light shaded at 30% and integrated with red and blue LED light at 1:1 (RB 1:1) and 2:1 (RB 2:1) ratios.

The total dry matter partitioning showed a higher harvest index in ‘Colomba’ compared to ‘Libra’, and similar values within each cultivar in all the lighting conditions (**Figure 4**).

### 3.2 Soluble sugars and starch, polyphenols and pigments in leaves

Cultivar (C) and light (L) influenced the soluble sugar content. Glucose was influenced in the vegetative phase (101 DAS) and at harvest (129 DAS) by C. Specifically, in ‘Libra’, glucose was 174% and 63% higher than in ‘Colomba’. In addition, at 101 DAS its content was positively influenced by L. RB 1:1 and RB 2:1 determined an increase of 92% and 56% of glucose compared to CNT as reported in **Table 3**. Instead, fructose and sucrose were influenced by C at 129 DAS. Their content was significantly higher in ‘Libra’ (+49% and +137%, respectively) than in ‘Colomba’. Sucrose was also influenced by the interaction C x L at 101 DAS. Particularly, sucrose increased in ‘Colomba’ under RB 1:1 by 41% compared to CNT, while in ‘Libra’, RB 1:1 and RB 2:1 determined a decrease of 25% and 28%, respectively, compared to CNT (**Table 3**). Polyphenols were significantly influenced only by C and only at 101 DAS. In fact, their content was higher in ‘Libra’ (on avg. 16.28 mg g^-1^ DW) than in ‘Colomba’ (13.66 mg g^-1^ DW). Finally, also pigments were significantly influenced by C, L and C x L. In particular, chlorophylls a (Chl a) and carotenoids at 101 DAS were higher in ‘Colomba’ (+18% and +19%, respectively) than in ‘Libra’ (**Table 3**). At 129 DAS, RB 1:1 and RB 2:1 determined a significant decrease in all pigments. Specifically, Chl a decreased by 24% and 41%, Chl b by 26% and 70% and carotenoids by 19% and 53%, respectively, compared to their CNT (**Table 3**). In ‘Colomba’, RB 2:1 decreased the content of Chl a, Chl b and carotenoids by 43%, 70% and 45%, respectively, compared to CNT; while in ‘Libra’ RB 1:1 and RB 2:1 decreased Chl a by 41% and 49%, respectively, while only RB 2:1 decreased carotenoids by 69% compared to CNT (**Table 3**).

**Table 3:** Starch and soluble sugars (in mg g^-1^ DW), polyphenols (in mg g^-1^ DW), chlorophylls and carotenoids (in mg g^-1^ DW) in leaves of potato plants cv. ‘Colomba’ and ‘Libra’ at vegetative phase (101 DAS) and at harvest (129 DAS) grown under natural light (control, CNT) and natural light shaded at 30% and integrated with red and blue LED light at 1:1 (RB 1:1) and 2:1 (RB 2:1) ratios.

Source of variance		Starch	Glucose	Fructose	Sucrose	Polyphenols	Chl a	Chl b	Carotenoids
Vegetative phase (101 DAS)									
Cultivar (C)									
	Colomba	6.41 ± 0.53	3.94 ± 0.38	10.76 ± 0.90	3.61 ± 0.28	13.66 ± 0.54	19.31 ± 0.37	4.67± 0.12	4.25 ± 0.10
	Libra	6.33 ± 0.88	9.43 ± 1.01	9.83 ± 0.47	3.44 ± 0.26	16.28 ± 0.43	16.45 ± 0.43	4.01 ± 0.49	3.59 ± 0.09
Light treatment (L)									
	CNT	7.11 ± 0.21	4.47 ± 0.75 a	10.01 ± 0.41	3.58 ± 0.03	14.69 ± 1.00	17.49 ± 0.91	4.36 ± 0.45	3.86 ± 0.23
	RB 1:1	5.56 ± 0.08	8.62 ± 0.30 b	10.83 ± 0.03	3.65 ± 0.09	15.17 ± 0.86	17.61 ± 0.51	4.47 ± 0.21	3.82 ± 0.11
	RB 2:1	6.44 ± 0.44	6.96 ± 0.29 b	10.03 ± 0.53	3.36 ± 0.02	15.05 ± 0.64	17.66 ± 0.89	4.19 ± 0.21	4.08 ± 0.19
C x L									
Colomba	CNT	7.18 ± 0.79	1.06 ± 0.04	10.13 ± 0.78	2.95 ± 0.22 b	12.71 ± 0.83	19.45 ± 0.47 ab	4.85 ± 0.11	4.35 ± 0.12 ab
	RB 1:1	6.31 ± 0.56	5.99 ± 0.72	11.51 ± 0.89	4.15 ± 0.26 a	14.25 ± 1.38	18.35 ± 0.72 ac	4.55 ± 0.04	3.95 ± 0.17 ac
	RB 2:1	5.74 ± 0.24	4.77 ± 0.39	10.63 ± 1.03	3.74 ± 0.31 ab	14.02 ± 0.45	20.15 ± 0.27 a	4.59 ± 0.22	4.44 ± 0.09 a
Libra	CNT	7.04 ± 1.09	7.89 ± 1.10	9.91 ± 0.20	4.21 ± 0.26 a	16.67 ± 0.65	15.52 ± 0.17 c	3.85 ± 0.87	3.36 ± 0.09 c
	RB 1:1	4.80 ± 0.68	11.25 ± 1.15	10.14 ± 0.93	3.14 ± 0.18 b	16.09 ± 0.97	16.86 ± 0.46 bc	4.39 ± 0.45	4.44 ± 0.12 c
	RB 2:1	7.14 ± 0.86	9.15 ± 0.80	9.44 ± 0.27	2.99 ± 0.34 b	16.07 ± 0.88	16.98 ± 1.16 bc	3.79 ± 0.14	3.72 ± 0.19 bc
Significance									
	Cultivar (C)	ns	***	ns	ns	**	***	ns	***
	Light treatment (L)	ns	***	ns	ns	ns	ns	ns	ns
	C x L	ns	ns	ns	**	ns	**	ns	***
At harvest (129 DAS)									
Cultivar (C)									
	Colomba	45.50 ± 4.54	17.10 ± 1.28	11.50 ± 1.10	1.39 ± 0.41	10.10 ± 0.64	8.15 ± 0.68	2.82 ± 0.38	2.87 ± 0.25
	Libra	41.02 ± 5.23	27.87 ± 2.82	17.10 ± 1.20	3.30 ± 0.59	10.15 ± 0.70	9.75 ± 0.61	3.09 ± 0.47	2.96 ± 0.31
Light treatment (L)									
	CNT	41.56 ± 1.39	24.85 ± 1.21	13.79 ± 0.51	2.78 ± 0.34	10.09 ± 0.54	11.52 ± 0.39 a	4.33 ± 0.07 a	3.86 ± 0.08 a
	RB 1:1	43.92 ± 4.09	19.99 ± 2.56	13.69 ± 1.41	1.91 ± 0.44	10.13 ± 0.61	9.17± 0.59 b	3.35 ± 0.08 b	3.27 ± 0.05 a
	RB 2:1	44.30 ± 4.03	22.63 ± 1.91	14.07 ± 0.20	2.35 ± 0.27	10.15 ± 0.75	6.16 ± 0.33 b	1.18 ± 0.05 c	1.62 ± 0.05 b
C x L									
Colomba	CNT	41.19 ± 2.40	20.29 ± 2.64	13.03 ± 2.76	2.15 ± 0.79	10.10 ± 0.64	9.14 ± 1.09 a	3.75 ± 0.59 a	3.39 ± 0.30 a
	RB 1:1	51.25 ± 1.24	13.54 ± 0.72	10.03 ± 1.17	0.80 ± 0.18	10.15 ± 0.76	10.09 ± 0.36 a	3.59 ± 0.40 ab	3.36± 0.23 a
	RB 2:1	44.07 ± 10.01	17.48 ± 0.48	11.45 ± 1.82	1.21 ± 0.26	10.09 ± 0.68	5.22 ± 0.58 c	1.13 ± 0.14 b	1.86 ± 0.22 b
Libra	CNT	41.92 ± 4.38	29.41 ± 0.93	17.47 ± 2.04	3.40 ± 0.30	10.15 ± 0.70	13.90 ± 0.53 b	4.91 ± 0.69 a	4.33 ± 0.43 a
	RB 1:1	36.60 ± 7.03	26.44 ± 4.34	17.14 ± 3.16	3.01 ± 0.81	10.19 ± 0.77	8.25 ± 1.20 a	3.11 ± 0.51 ab	3.19 ± 0.31 a
	RB 2:1	44.54 ± 4.30	27.78 ± 3.19	16.69 ± 1.79	3.49 ± 0.65	10.21 ± 1.04	7.11 ± 0.10 a	1.24 ± 0.22 b	1.37 ± 0.18 b
Significance									
	Cultivar (C)	ns	***	**	**	ns	ns	ns	ns
	Light treatment (L)	ns	ns	ns	ns	ns	***	***	***
	C x L	ns	ns	ns	ns	ns	**	**	***

Mean values ± standard errors; n=3. Different letters indicate significant differences according to Tukey’s multiple test (p<0.05). ns: not significant; ** p<0.01; *** p<0.001.

### 3.3 Soluble proteins and free amino acids in leaves

The soluble protein content in leaves was influenced at 101 DAS by L, C and C x L. As reported in **Table 4**, its content was higher in ‘Colomba’ than in ‘Libra’ (on avg. 158.62 mg g^-1^ DW and 146.39 mg g^-1^, DW respectively) and the light treatment RB 2:1 increased it by 7% compared to CNT (**Table 4**). However, C x L negatively affected ‘Colomba’ under RB 1:1, with a decrease in protein content of 9% compared to CNT (**Table 4**).

**Table 4:** Soluble proteins (in mg g^-1^ DW) and free amino acids (in µmol g^-1^ DW) in leaves of potato plants cv. ‘Colomba’ and ‘Libra’ at vegetative phase (101 DAS) and at harvest (129 DAS) grown under natural light (control, CNT) and natural light shaded at 30% and integrated with red and blue LED light at 1:1 (RB 1:1) and 2:1 (RB 2:1) ratios.

	Cultivar (C)		Light treatment (L)			C x L						Significance		
	Colomba	Libra	CNT	RB 1:1	RB 2:1	Colomba - CNT	Colomba - RB 1:1	Colomba - RB 2:1	Libra - CNT	Libra - RB 1:1	Libra - RB 2:1	Cultivar	Light treatment	C x L
Vegetative phase (101 DAS)														
Proteins	158.62 ± 3.68	146.39 ± 2.39	149.89 ± 4.93 b	146.79 ± 3.00 b	160.84 ± 3.69 a	160.73 ± 1.02 ab	146.50 ± 5.64 bc	168.63 ± 2.07 a	139.05 ± 1.76 c	147.08 ± 3.61 bc	153.05 ± 1.77 bc	***	**	**
Ala	6.14 ± 0.62	4.57 ± 0.29	4.35 ± 0.32	5.64 ± 0.69	6.08 ± 0.76	4.30 ± 0.61	6.88 ± 0.68	7.25 ± 1.08	4.39 ± 0.36	4.41 ± 0.63	4.91 ± 0.60	*	ns	ns
Arg	0.83 ± 0.18	0.67 ± 0.09	0.45 ± 0.15 b	0.86 ± 0.17 ab	0.96 ± 0.14 a	0.27 ± 0.22	1.16 ± 0.14	1.07 ± 0.27	0.63 ± 0.16	0.55 ± 0.18	0.84 ± 0.11	ns	*	ns
Asn	1.68 ± 0.39	2.16 ± 0.53	2.59 ± 0.78	1.15 ± 0.29	2.01 ± 0.40	2.21 ± 1.06	1.18 ± 0.18	1.64 ± 0.63	2.97 ± 1.33	1.12 ± 0.62	2.38 ± 0.51	ns	ns	ns
Asp	8.97 ± 1.09	5.22 ± 0.30	5.60 ± 0.65 b	6.90 ± 1.08 ab	8.80 ± 1.59 a	6.46 ± 1.06	8.64 ± 1.64	11.81 ± 1.77	4.74 ± 0.48	5.15 ± 0.30	5.78 ± 0.70	**	*	ns
GABA	4.80 ± 0.55	3.51 ± 0.24	3.63 ± 0.33	4.62 ± 0.73	4.22 ± 0.60	3.56 ± 0.69	6.04 ± 0.70	4.81 ± 1.03	3.70 ± 0.23	3.19 ± 0.40	3.64 ± 0.63	*	ns	ns
Gln	8.26 ± 1.58	3.59 ± 0.49	4.09 ± 0.79 b	4.80 ± 1.03 ab	8.89 ± 2.33 a	5.22 ± 1.24	6.60 ± 1.10	12.97 ± 3.15	2.97 ± 0.59	3.00 ± 0.95	4.80 ± 0.76	**	*	ns
Glu	45.53 ± 4.28	23.16 ± 1.49	28.06 ± 4.64	37.14 ± 6.46	37.83 ± 7.02	34.21 ± 7.51	50.27 ± 5.34	52.11 ± 6.14	21.91 ± 3.64	24.01 ± 2.74	23.55 ± 2.16	***	ns	ns
Gly	8.34 ± 1.50	6.18 ± 0.54	4.38 ± 0.93 b	7.30 ± 0.86 ab	10.11 ± 1.39 a	3.53 ± 1.68	8.86 ± 0.90	12.64 ± 1.58	5.24 ± 0.87	5.74 ± 0.68	7.57 ± 0.89	*	***	ns
Met	0.16 ± 0.03	0.14 ± 0.02	0.14 ± 0.04	0.16 ± 0.03	0.17 ± 0.02	0.15 ± 0.05	0.18 ± 0.02	0.16 ± 0.02	0.14 ± 0.02	0.13 ± 0.03	0.17 ± 0.02	ns	ns	ns
Pro	9.57 ± 1.28	4.48 ± 0.41	7.07 ± 1.62	5.11 ± 0.76	8.89 ± 1.90	10.34 ± 1.54	6.41 ± 1.00	11.96 ± 2.88	3.80 ± 0.24	3.82 ± 0.47	5.83 ± 0.59	***	ns	ns
Ser	6.32 ± 0.80	2.95 ± 0.46	3.55 ± 0.73	4.63 ± 1.08	5.72 ± 1.25	4.46 ± 1.20	6.56 ± 0.83	7.93 ± 1.58	2.64 ± 0.64	2.71 ± 1.19	3.50 ± 0.66	**	ns	ns
Tyr	1.29 ± 0.13	0.69 ± 0.10	0.77 ± 0.19	1.03 ± 0.15	1.19 ± 0.21	1.05 ± 0.18	1.30 ± 0.17	1.52 ± 0.30	0.48 ± 0.25	0.76 ± 0.11	0.85 ± 0.11	**	ns	ns
Total AA	116.49 ± 10.70	68.79 ± 5.11	77.81 ± 8.59	92.01 ± 13.89	108.10 ± 17.89	90.01 ± 10.31	118.83 ± 12.31	140.62 ± 21.71	65.60 ± 10.68	65.20 ± 9.69	75.58 ± 8.45	***	ns	ns
Essential AA	10.17 ± 0.65	7.37 ± 0.71	8.42 ± 0.95	8.36 ± 1.00	9.55 ± 1.15	9.38 ± 1.03	10.10 ± 0.56	11.04 ± 1.75	7.45 ± 1.59	6.62 ± 1.30	8.05 ± 1.13	*	ns	ns
BCAAs	2.51 ± 0.21	2.45 ± 0.19	2.29 ± 0.29	2.50 ± 0.20	2.65 ± 0.23	2.22 ± 0.45	2.63 ± 0.28	2.69 ± 0.40	2.36 ± 0.46	2.37 ± 0.32	2.61 ± 0.31	ns	ns	ns
At harvest (129 DAS)														
Proteins	15.61 ± 2.43	14.72 ± 1.91	15.16 ± 1.19	16.20 ± 1.60	21.29 ± 2.80	15.61 ± 2.43	18.75 ± 2.71	17.99 ± 2.75	14.72 ± 1.91	13.13 ± 1.60	24.60 ± 3.87	ns	ns	ns
Ala	7.47 ± 1.17	5.17 ± 0.69	6.30 ± 0.89	6.32 ± 0.95	6.15 ± 0.98	7.47 ± 1.17	6.88 ± 0.87	6.68 ± 0.86	5.17 ± 0.69	5.45 ± 0.71	4.89 ± 0.82	***	ns	ns
Arg	0.85 ± 0.11	0.71 ± 0.09	0.87 ± 0.10 a	0.82 ± 0.09 ab	0.70 ± 0.09 b	0.85 ± 0.11	0.82 ± 0.09	0.75 ± 0.10	0.71 ± 0.09	0.67 ± 0.08	0.59 ± 0.07	**	*	ns
Asn	11.34 ± 2.16	2.75 ± 0.57	9.77 ± 1.88	8.42 ± 1.78	8.63 ± 1.79	11.34 ± 2.16	10.50 ± 1.89	9.70 ± 1.85	2.75 ± 0.57	2.42 ± 0.51	2.56 ± 0.52	***	ns	ns
Asp	15.83 ± 2.55	8.23 ± 1.12	14.49 ± 2.28	13.59 ± 2.11	12.43 ± 2.05	15.83 ± 2.55	14.90 ± 2.20	14.43 ± 2.25	8.23 ± 1.12	8.09 ± 1.17	8.36 ± 1.21	***	ns	ns
GABA	45.65 ± 7.47	22.01 ± 3.63	39.50 ± 6.24	35.00 ± 5.95	38.92 ± 6.14	45.65 ± 7.47	40.50 ± 6.33	43.28 ± 6.50	22.01 ± 3.63	20.00 ± 3.39	24.15 ± 3.84	**	ns	ns
Gln	17.08 ± 3.34	8.60 ± 1.58	12.00 ± 2.51 a	13.80 ± 2.67 a	16.62 ± 3.18 b	17.08 ± 3.34	20.63 ± 3.85	18.20 ± 3.65	8.60± 1.58	8.80 ± 1.73	8.40 ± 1.59	*	***	ns
Glu	0.36 ± 0.04	0.29 ± 0.03	0.34 ± 0.03 a	0.35 ± 0.04 ab	0.29 ± 0.03 b	0.36 ± 0.04	0.32 ± 0.03	0.34 ± 0.03	0.29 ± 0.03	0.30 ± 0.03	0.27 ± 0.03	*	*	ns
Gly	0.22 ± 0.03	0.29 ± 0.04	0.21 ± 0.03 b	0.22 ± 0.03 b	0.28 ± 0.04 a	0.22 ± 0.03 b	0.23 ± 0.03 b	0.21 ± 0.03 b	0.29 ± 0.04 b	0.28 ± 0.04 b	0.30 ± 0.04 a	**	**	**
Met	0.7 ± 0.12 a	0.51 ± 0.03 b	0.67 ± 0.07	0.61 ± 0.07	0.54 ± 0.17	0.68 ± 0.12	0.76 ± 0.13	0.66 ± 0.11	0.65 0.024	0.47 ± 0.01	0.43 ± 0.08	*	ns	ns
Pro	10.23 ± 1.24	5.78 ± 0.71	9.00 ± 1.09	8.62 ± 1.03	9.33 ± 1.10	10.23 ± 1.24	9.88 ± 1.19	9.45 ± 1.14	5.78 ± 0.71	5.67 ± 0.69	5.89 ± 0.72	**	ns	ns
Ser	0.25 ± 0.04	0.20 ± 0.03	0.24 ± 0.04	0.22 ± 0.03	0.21 ± 0.03	0.25 ± 0.04	0.23 ± 0.03	0.24 ± 0.03	0.20 ± 0.03	0.20 ± 0.03	0.19 ± 0.03	**	ns	ns
Tyr	0.07 ± 0.01	0.05 ± 0.01	0.07 ± 0.01	0.07 ± 0.01	0.06 ± 0.01	0.07 ± 0.01	0.07 ± 0.01	0.06 ± 0.01	0.05 ± 0.01	0.05 ± 0.01	0.05 ± 0.01	***	ns	ns
Total AA	133.16 ± 11.96	80.57 ± 3.50	105.50 ± 20.35	120.88 ± 27.96	94.22 ± 16.09	130.41 ± 17.39	155.12 ± 21.79	113.93 ± 13.19	80.57 ± 5.18	86.63 ± 3.55	74.51 ± 9.95	***	ns	ns
Essential AA	12.37 ± 1.83	8.22 ± 1.21	11.52 ± 1.66	10.62 ± 1.52	9.97 ± 1.43	12.37 ± 1.83	12.28 ± 1.80	12.04 ± 1.75	8.22 ± 1.21	8.10 ± 1.19	8.14 ± 1.20	**	ns	ns
BCAAs	7.72 ± 1.18	5.49 ± 0.34	7.68 ± 0.67 a	7.06 ± 0.82 a	5.09 ± 0.82 b	7.94 ± 1.10	8.72 ± 1.42	6.52 ± 1.03	7.41 ± 0.21	5.4 ± 0.21	3.66 ± 0.61	**	*	ns

Mean values ± standard errors; n=3. Different letters indicate significant differences according to Tukey’s multiple test (p<0.05). ns: not significant; * p<0.05; ** p<0.01; *** p<0.001.

At 101 DAS and 129 DAS, total amino acids content was affected by C, with ‘Colomba’ showing the highest values (+71% and + 66%, respectively, compared to ‘Libra’) (**Table 4**). Among these amino acids, in ‘Colomba’ there was a higher concentration of essential amino acids (+38% and 50%, respectively), especially arginine (+24% and +20%) compared to ‘Libra’. Also, at 129 DAS, C and L significantly influenced the branched-chain amino acids (BCAAs) content, which was higher in ‘Colomba’ (on avg. 7.72 µmol g^-1^ DW) than in ‘Libra’ (on avg. 5.49 µmol g^-1^ DW). Additionally, as reported in **Table 4**, in ‘Colomba’ at 101 DAS there was a higher content of alanine, aspartate, GABA, glutamine, proline, serine and tyrosine compared to ‘Libra’, by 34%, 72%, 37%, 130%, 114%, 114% and 87%, respectively; while at 129 DAS it was higher than that of ‘Libra’ by 44%, 92%, 107%, 98%, 77%, 25% and 40% % respectively and. Also, glycine was influenced by C. Particularly, at 101 DAS it was higher in ‘Colomba’ (on avg. 8.34 µmol g^-1^ DW) than in ‘Libra’ (on avg. 6.18 µmol g^-1^ DW); while at harvest it was lower in ‘Colomba’ (on avg. 0.22 µmol g^-1^ DW) than in ‘Libra’ (on avg. 0.29 µmol g^-1^ DW). Light treatment (L) also influenced some amino acids at 101 DAS and 129 DAS. In particular, at 101 DAS, RB 2:1 determined an increase in arginine, aspartate, glutamine and glycine of 113%, 57%, 117% and 131%, respectively, compared to CNT; while at 129 DAS RB 2:1 it enhanced glutamine (+39%) and glycine (+33%) compared to CNT, while decreased arginine (-20%) and glutamate (-15%) compared to respective CNT (**Table 4**).

### 3.4 Principal component analysis on leaves

**Figure 5:**
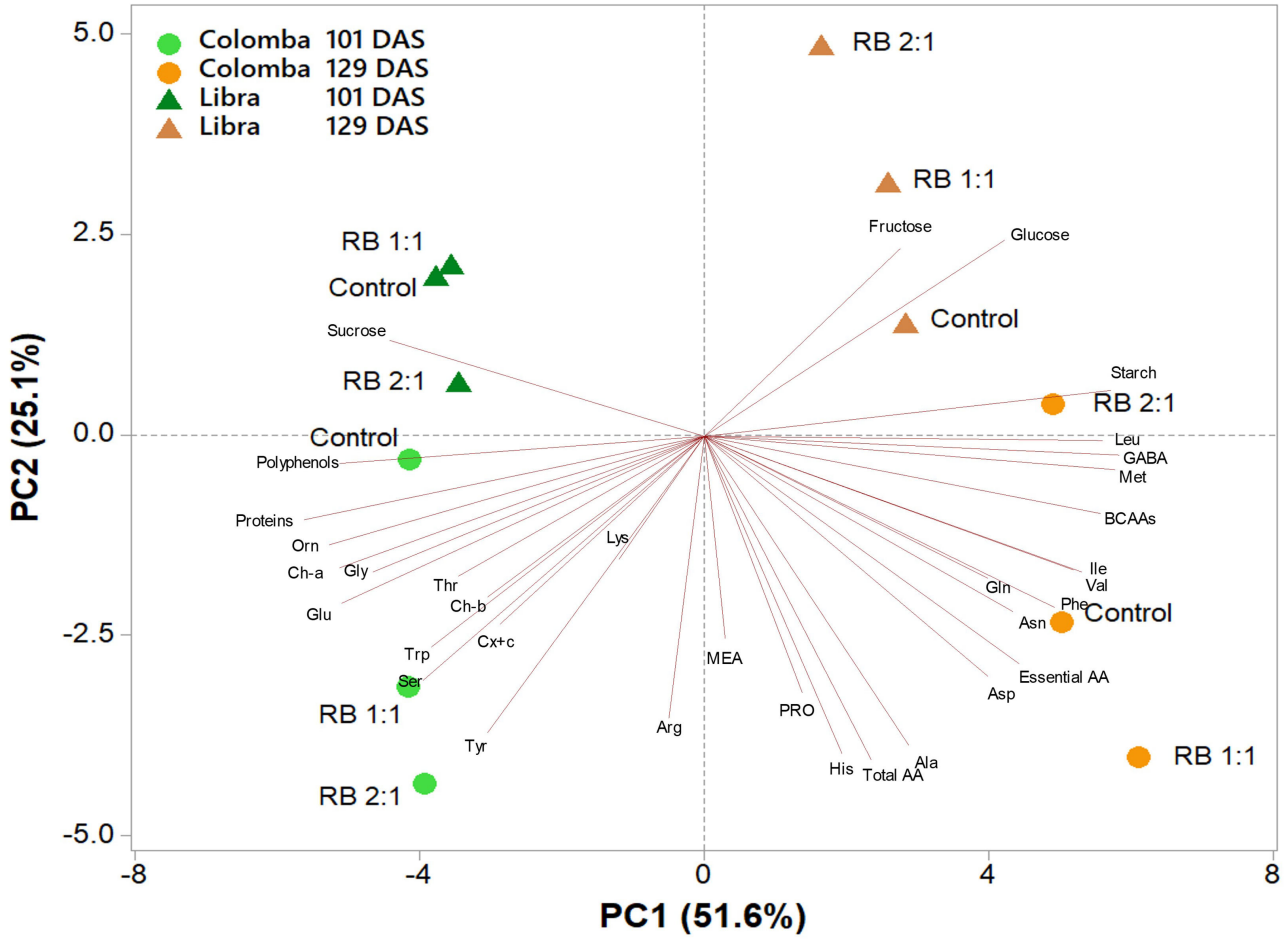
Principal component analysis (PCA) loading plot and scores of carbohydrates, amino acids, photosynthetic pigments, polyphenols, and soluble proteins in leaves of potato cultivars ‘Libra’ and ‘Colomba’ grown under natural light (control, CNT) and natural light shaded at 30% and integrated with red and blue LED light at 1:1 (RB 1:1) and 2:1 (RB 2:1) ratios, at two vegetative stages (101 and 129 DAS).

A principal component analysis (PCA) was performed on all metabolites determined in leaf tissues of potato cultivars ‘Libra’ and ‘Colomba’ at two vegetative stages (101 and 129 DAS) and subjected to three light treatments (Control, RB 1:1, and RB 2:1). The variables in the first four principal components (PCs) were highly correlated, with eigenvalues higher than 1, explaining 89.9% of the total variance, with PC1, PC2, PC3, and PC4 accounting for 51.6%, 25.1%, 7.0%, and 6.1%, respectively. The two cultivars at different vegetative stages were well separated along PC1, with those at 101 DAS and 129 DAS clustered on the negative and positive sides of PC1, respectively. The ‘Colomba’ samples were clustered on the negative side of PC2, while ‘Libra’ ones were clustered on the positive side of PC2. PC1 was positively correlated to GABA, methionine, starch, BCAAs (leucine, valine and isoleucine), and phenylalanine, whereas it was negatively correlated to proteins, ornithine, polyphenols, chlorophyll a, and glutamate. PC2 was positively correlated to fructose and glucose, while it was negatively correlated to total amino acids, histidine, alanine, tyrosine, arginine, proline, serine and aspartate. This indicates that metabolites are differentially regulated in ‘Libra’ and ‘Colomba’, with ‘Libra’ having higher levels of soluble carbohydrates (in particular, glucose and fructose) and lower levels of amino acids, which are mainly concentrated in ‘Colomba’. At least on leaves, light treatments do not seem to have a main role. The PCA plot shows, in fact, distinct clustering patterns for the different light treatments, but the effects are not uniform across all samples. As an example, GABA, BCAAs, and starch show different responses to RB 1:1 and RB 2:1 treatments depending on the cultivar and vegetative stage (**Figure 5**).

### 3.5 Potato tubers mineral profile

The concentration of the main minerals in potato tubers is provided in **Table 5**. According to the assessed mineral profile, K is the most abundant element (19.6-22.6 g kg^-1^ DW) in the potato tubers; these latter are also an important source of P (2.1-2.3 g kg^-1^ DW), Mg (1.2-1.5 g kg^-1^ DW) and Ca (172-413 mg kg^-1^ DW), and other essential elements, such as Na, Fe, Zn, Mn, B and Cu, present in concentrations lower than 0.1 g kg^-1^ DW (**Table 3**). We found statistically significant differences in the mineral concentrations in tubers of two cultivars. The cv. ‘Colomba’, in fact, showed significantly higher concentrations of all the minerals (except P and Cu) in its tubers than the cv. ‘Libra’ (**Table 5**), probably due to the different genomic traits and the tendentially lower tuber biomass production (see **Table 2**). On the other hand, the effect of supplemental LED lighting with RB 1:1 and RB 2:1 did not produce statistically significant differences in terms of mineral concentrations in potato tubers, in comparison with the CNT. Nevertheless, the Zn concentration in the tubers of plants treated with RB 2:1 was significantly higher than that of plants treated with RB 1:1, while the opposite trend was observed for Cu concentration (**Table 5**). In none of the minerals, the interaction between light treatments and cultivars was statistically significant.

**Table 5:** Concentration (g kg^-1^ or mg kg^-1^ DW) of main nutrients in tubers of potato cv. ‘Colomba’ and ‘Libra’ grown under natural light (control, CNT) and natural light shaded at 30% and integrated with red and blue LED light at 1:1 (RB 1:1) and 2:1 (RB 2:1) ratios.

		K	P	Mg	Ca	Na	Fe	Zn	Mn	B	Cu
		g kg^-1^ DW			mg kg^-1^ DW						
Colomba	CNT	22.6 ± 1.0	2.2 ± 0.1	1.5 ± 0.1	329 ± 34	62.9 ± 6.3	20.7 ± 1.7	6.7 ± 0.4	7.1 ± 0.4	5.8 ± 0.2	3.3 ± 0.4
	RB 1:1	20.9 ± 1.0	2.1 ± 0.1	1.3 ± 0.1	323 ± 26	50.0 ± 2.6	23.7 ± 2.5	6.3 ± 0.4	6.3 ± 0.4	5.9 ± 0.2	3.5 ± 0.3
	RB 2:1	22.1 ± 0.6	2.3 ± 0.1	1.4 ± 0.1	413 ± 38	63.1 ± 5.5	22.6 ± 1.0	7.7 ± 0.3	6.7 ± 0.2	5.7 ± 0.1	2.8 ± 0.3
Libra	CNT	19.8 ± 0.5	2.2 ± 0.1	1.2 ± 0.1	172 ± 10	42.2 ± 1.3	14.7 ± 0.7	6.1 ± 0.2	4.7 ± 0.1	5.3 ± 0.1	4.0 ± 0.3
	RB 1:1	19.7 ± 0.7	2.2 ± 0.1	1.2 ± 0.1	206 ± 7	42.9 ± 1.1	17.1 ± 0.7	5.7 ± 0.2	4.7 ± 0.2	5.6 ± 0.3	4.5 ± 0.3
	RB 2:1	19.6 ± 0.3	2.3 ± 0.1	1.2 ± 0.1	186 ± 9	39.4 ± 1.8	16.9 ± 0.4	6.3 ± 0.2	4.9 ± 0.1	5.4 ± 0.1	3.4 ± 0.2
Significance											
Cultivar (C)		**	ns	***	***	***	***	**	***	*	**
Light treatment (L)		ns	ns	ns	ns	ns	ns	*	ns	ns	*
C x L		ns	ns	ns	ns	ns	ns	ns	ns	ns	ns

Mean values ± Standard Errors; n=6. Different letters within each column indicate significant differences according to Tukey’s multiple-range test (p<0.05). ns, not significant; * p<0.05; ** p<0.01; *** p<0.001.

### 3.6 Soluble sugars and starch, polyphenols and glycoalkaloids in potato tubers

In tubers, C influenced soluble sugars content. In particular, the content of glucose was higher in ‘Colomba’ (+46%) than in ‘Libra’; while fructose was lower in ‘Colomba’ (-12%) compared to ‘Libra’ (**Table 6**). Also, the polyphenol content was affected by C, and the highest values were in ‘Colomba’ (+13%) than in Libra. C and L also influenced the glycoalkaloids α-solanine and α-chaconine. As reported in **Table 6**, both the glycoalkaloids were 39% and 15% higher in ‘Colomba’ than in ‘Libra’. RB 2:1 induced a decrease in α-solanine of 56% compared to CNT; whereas α-chaconine was decreased under RB 1:1 and RB 2:1 by 36% and 35%, respectively, compared to CNT (**Table 6**).

**Table 6:** Starch and soluble sugars (in mg g^-1^ DW), polyphenols (in mg g^-1^ DW), and glycoalkaloids (in mg kg^-1^ DW) in tubers of potato plants cv. ‘Colomba’ and ‘Libra’ grown under natural light (control, CNT) and natural light shaded at 30% and integrated with red and blue LED light at 1:1 (RB 1:1) and 2:1 (RB 2:1) ratios.

Source of variance		Starch	Glucose	Fructose	Sucrose	Polyphenols	α-solanine	α-chaconine
Cultivar (C)								
	Colomba	48.34 ± 1.52	20.43 ± 1.47 a	9.70 ± 0.59 a	3.89 ± 0.18	2.90 ± 0.07 a	79.43 ± 11.4	126.86 ± 12.0
	Libra	49.37 ± 0.42	13.98 ± 1.00 b	10.90 ± 0.33 b	3.73 ± 0.18	2.55 ± 0.09 b	56.92 ± 7.10	110.45 ± 8.81
Light treatment (L)								
	CNT	48.83 ± 1.55	18.38 ± 0.61	10.20 ± 0.36	3.96 ± 0.06	2.65 ± 0.18	84.17 ± 1.32 a	156.55 ± 9.51 a
	RB 1:1	48.65 ± 1.13	17.52 ± 0.35	10.32 ± 0.05	3.90 ± 0.06	2.86 ± 0.08	83.70 ± 14.3 a	96.66 ± 4.80 b
	RB 2:1	49.09 ± 0.64	15.73 ± 0.05	10.38 ± 0.13	3.58 ± 0.02	2.66 ± 0.05	36.66 ± 1.95 b	102.76 ± 4.85 b
C x L								
Colomba	CNT	48.74 ± 1.85	22.10 ± 1.47	9.25 ± 0.74	3.98 ± 0.19	2.93 ± 0.20	84.98 ± 2.84 b	172.67 ± 5.34
	RB 1:1	48.33 ± 1.65	21.90 ± 1.89	9.78 ± 0.68	4.06 ± 0.24	3.03 ± 0.05	115.45 ± 4.07 a	104.36 ± 3.49
	RB 2:1	47.95 ± 1.05	17.29 ± 1.06	10.06 ± 0.34	3.63 ± 0.10	2.74 ± 0.01	37.88 ± 2.62 d	103.55 ± 10.4
Libra	CNT	48.91 ± 0.45	14.65 ± 0.60	11.14 ± 0.23	3.94 ± 0.09	2.37 ± 0.22	83.36 ± 0.07 b	140.43 ± 12.8
	RB 1:1	48.98 ± 0.61	13.13 ± 1.40	10.87 ± 0.62	3.73 ± 0.32	2.70 ± 0.07	51.96 ± 1.25 c	88.96 ± 6.60
	RB 2:1	50.22 ± 0.22	14.16 ± 0.99	10.70 ± 0.15	3.51 ± 0.13	2.59 ± 0.09	35.45 ± 3.27 d	101.96 ± 2.94
Significance								
Cultivar (C)		ns	***	*	ns	**	***	*
Light treatment (L)		ns	Ns	ns	ns	ns	***	***
C x L		ns	Ns	ns	ns	ns	***	ns

Mean values ± standard errors; n=3.

Different letters indicate significant differences according to Tukey’s multiple test (p<0.05). ns, not significant; * p<0.05; ** p<0.01; *** p<0.001.

### 3.7 Soluble proteins and free amino acids in potato tubers

Total and essential amino acids were influenced by C, with higher values in ‘Colomba’ (+102% and +30%, respectively) than in ‘Libra’. In addition, arginine, asparagine, aspartate, glutamine, glycine, ornithine, proline, serine and tyrosine were 148%, 170%, 48%, 91%, 79%, 14%, 65%, 58% and 171% higher in ‘Colomba’ than in ‘Libra’, respectively (**Table 7**); while in ‘Libra’ alanine, GABA and monoethanolamine (MEA) were 34%, 81% and 41% respectively higher in ‘Libra’ than in ‘Colomba’ (**Table 7**).

**Table 7:** Soluble proteins (in mg g^-1^ DW) and free amino acids (in µmol g^-1^ DW) in tubers of potato plants cv. ‘Colomba’ and ‘Libra’ grown under natural light (control, CNT) and natural light shaded at 30% and integrated with red and blue LED light at 1:1 (RB 1:1) and 2:1 (RB 2:1) ratios.

	Cultivar (C)		Light treatment (L)			C x L							Significance	
	Colomba	Libra	CNT	RB 1:1	RB 2:1	Colomba x CNT	Colomba x RB 1:1	Colomba x RB 2:1	Libra x CNT	Libra x RB 1:1	Libra x RB 2:1	Cultivar	Light treatment	C x L
Proteins	33.90 ± 1.96	32.82 ± 1.48	34.16 ± 1.54	33.52 ± 3.19	32.39 ± 1.35	36.66 ± 1.42	35.12 ± 5.40	29.91 ± 1.62	31.65 ± 1.90	31.92 ± 4.39	34.88 ± 0.51	ns	ns	ns
Ala	2.11 ± 0.13	2.82 ± 0.25	2.67 ± 0.32	2.57 ± 0.32	2.17 ± 0.18	2.26 ± 0.22	2.12 ± 0.15	1.97 ± 0.35	3.08 ± 0.55	3.02 ± 0.53	2.37 ± 0.07	*	ns	ns
Arg	4.57 ± 0.32	1.84 ± 0.13	3.55 ± 0.82	3.36 ± 0.64	2.71 ± 0.48	5.38 ± 0.19	4.62 ± 0.60	3.72 ± 0.37	1.72 ± 0.16	2.10 ± 0.34	1.70 ± 0.14	***	ns	ns
Asn	170.70 ± 14.66	63.43 ± 2.60	133.83 ± 31.76	115.02 ± 28.19	102.35 ± 18.84	200.59 ± 23.93	170.51 ± 29.82	141.01 ± 15.16	67.06 ± 3.57	59.53 ± 2.07	63.69 ± 7.07	***	ns	ns
Asp	5.27 ± 0.35	3.56 ± 0.12	4.47 ± 0.44	4.35 ± 0.58	4.42 ± 0.49	5.25 ± 0.56	5.47 ± 0.62	5.07 ± 0.86	3.68 ± 0.21	3.23 ± 0.13	3.77 ± 0.20	***	ns	ns
GABA	6.56 ± 0.40	11.90 ± 0.99	10.57 ± 1.79	8.62 ± 1.23	8.50 ± 1.33	7.30 ± 0.48	6.65 ± 0.34	5.72 ± 0.97	13.84 ± 2.26	10.59 ± 1.89	11.27 ± 0.43	***	ns	ns
Gln	5.03 ± 0.48	2.63 ± 0.28	4.40 ± 0.80	3.94 ± 0.61	3.14 ± 0.66	5.90 ± 0.86	4.82 ± 0.92	4.36 ± 0.76	2.91 ± 0.48	3.05 ± 0.49	1.92 ± 0.34	***	ns	ns
Glu	11.72 ± 0.61	6.54 ± 0.52	9.47 ± 1.22	9.32 ± 1.56	8.60 ± 1.24	11.52 ± 1.29	12.30 ± 1.48	11.32 ± 0.52	7.43 ± 1.24	6.33 ± 1.05	5.88 ± 0.13	***	ns	ns
Gly	5.69 ± 0.32	3.18 ± 0.19	4.68 ± 0.70	4.52 ± 0.69	4.10 ± 0.53	6.09 ± 0.45	5.92 ± 0.49	5.04 ± 0.70	3.27 ± 0.50	3.11 ± 0.38	3.17 ± 0.18	***	ns	ns
MEA	0.34 ± 0.02	0.48 ± 0.03	0.41 ± 0.04	0.43 ± 0.05	0.38 ± 0.04	0.34 ± 0.02	0.36 ± 0.03	0.30 ± 0.05	0.48 ± 0.07	0.50 ± 0.09	0.45 ± 0.02	**	ns	ns
Met	2.23 ± 0.15 a	1.37 ± 0.18 b	2.02 ± 0.2	1.83 ± 0.18	1.56 ± 0.09	2.49 ± 0.16 a	2.20 ± 0.12 ab	2.02 ± 0.16 ab	1.54 ± 0.26 bc	1.46 ± 0.25 bc	1.11 ± 0.02 c	***	ns	**
Orn	1.21 ± 0.05	1.06 ± 0.06	1.23 ± 0.07	1.17 ± 0.06	1.02 ± 0.05	1.33 ± 0.11	1.20 ± 0.05	1.12 ± 0.06	1.14 ± 0.08	1.14 ± 0.12	0.92 ± 0.00	*	ns	ns
Pro	6.03 ± 0.36	3.65 ± 0.21	5.27 ± 0.73	4.83 ± 0.48	4.41 ± 0.65	6.59 ± 0.83	5.82 ± 0.37	5.67 ± 0.70	3.95 ± 0.52	3.84 ± 0.22	3.15 ± 0.13	***	ns	ns
Ser	2.20 ± 0.11	1.39 ± 0.11	1.84 ± 0.24	1.88 ± 0.21	1.68 ± 0.23	2.26 ± 0.24	2.21 ± 0.20	2.13 ± 0.22	1.41 ± 0.24	1.55 ± 0.26	1.22 ± 0.03	***	ns	ns
Tyr	5.32 ± 0.38	1.96 ± 0.19	4.18 ± 1.04	3.70 ± 0.64	3.03 ± 0.71	6.38 ± 0.69	5.00 ± 0.53	4.57 ± 0.34	1.97 ± 0.35	2.41 ± 0.27	1.49 ± 0.10	***	ns	ns
Total AA	258.05 ± 17.93	127.38 ± 5.36	216.21 ± 38.06	192.10 ± 33.61	169.83 ± 24.67	294.90 ± 29.58	258.51 ± 34.68	220.74 ± 20.15	137.52 ± 13.22	125.69 ± 6.04	118.91 ± 6.66	***	ns	ns
Essential AA	35.88 ± 1.60	24.77 ± 2.19	33.20 ± 3.50	31.75 ± 2.95	26.04 ± 3.08	39.08 ± 2.58	36.11 ± 2.68	32.46 ± 2.43	27.32 ± 4.49	27.39 ± 4.16	19.61 ± 0.47	***	ns	ns
BCAAs	16.31 ± 0.59	15.01 ± 1.36	17.13 ± 1.29	16.47 ± 1.23	13.37 ± 0.87	17.46 ± 0.88	16.45 ± 0.89	15.00 ± 1.01	16.81 ± 2.73	16.49 ± 2.59	11.75 ± 0.30	ns	ns	ns

Mean values ± standard errors; n=3. Different letters indicate significant differences according to Tukey’s multiple test (p<0.05). ns, not significant; * p<0.05; ** p<0.01; *** p<0.001.

### 3.8 Principal component analysis on tuberous

**Figure 6:**
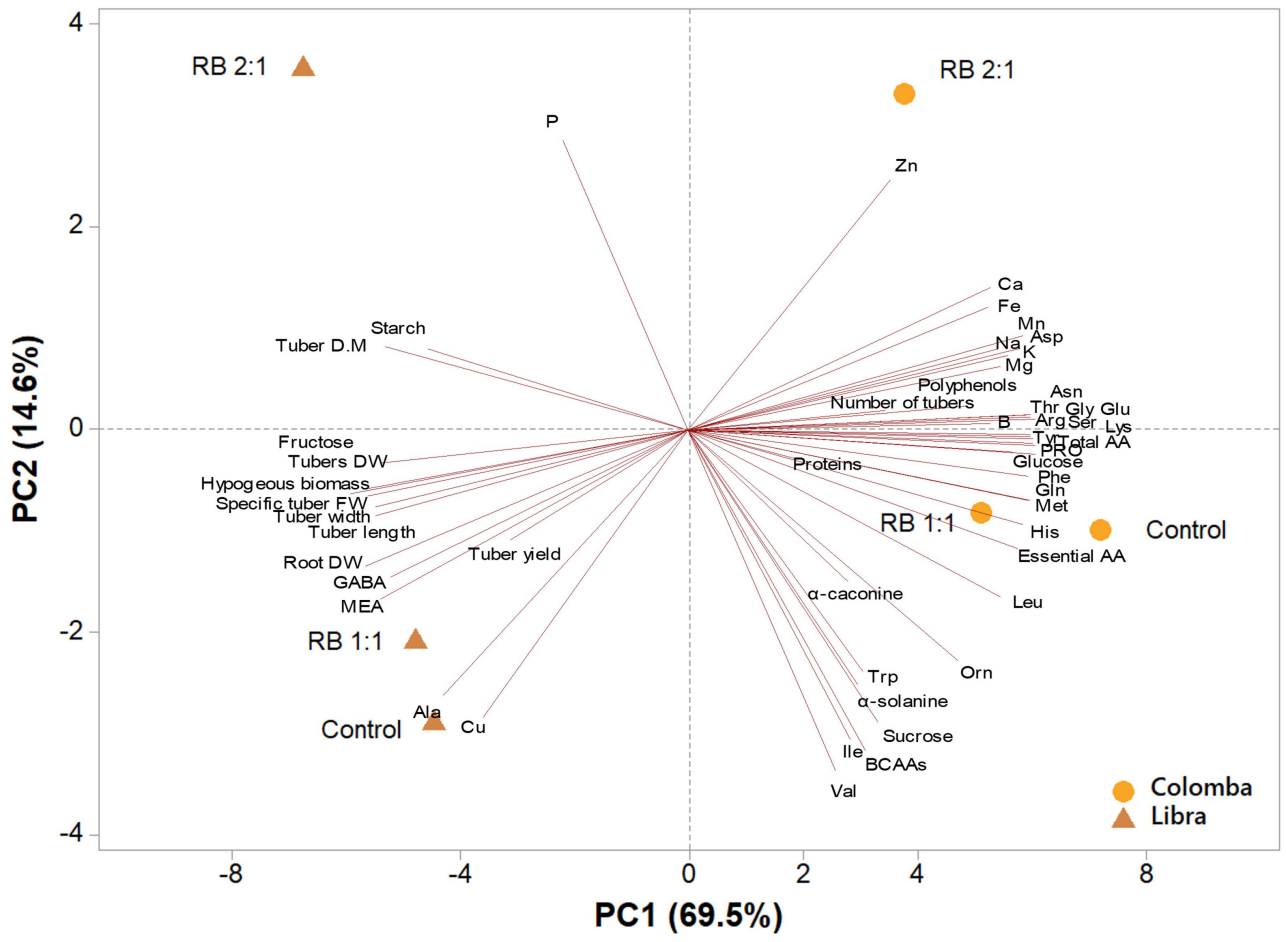
Principal component analysis (PCA) loading plot and scores of morphometric traits and metabolites in the tubers of potato cultivars ‘Libra’ and ‘Colomba’, grown under natural light (control, CNT) and natural light shaded at 30% and integrated with red and blue LED light at 1:1 (RB 1:1) and 2:1 (RB 2:1) ratios.

A second PCA was performed. A principal component analysis (PCA) was performed on all parameters analysed in tuber tissues of potato cultivars ‘Libra’ and ‘Colomba’, subjected to the three light treatments (CNT, RB 1:1, and RB 2:1) at harvest. The variables in the first four principal components (PCs) were highly correlated, with eigenvalues higher than 1, explaining 97.5% of the total variance. Specifically, PC1 accounted for 69.5%, PC2 for 14.6%, PC3 for 8.3%, and PC4 for 5.0% of the variance. The analysis highlighted a clear separation based on both cultivar and light treatment. The two cultivars were distinctly separated along PC1, with ‘Colomba’ and ‘Libra’ clustered on the positive and negative sides of PC1, respectively. PC1 was positively correlated to total amino acids, essential amino acids (in particular arginine, histidine, lysine, threonine, methionine and phenylalanine), and other primary amino acids (such as proline, tyrosine, serine, glycine, and asparagine), in addition to glucose, K and Mg; whereas, PC2 was negatively correlated to tuber FW, width, length, DW and DM, hypogeous biomass, fructose and MEA. PC2 was negatively correlated to valine, BCAAs, isoleucine, sucrose, Cu, alanine and α-solanine, while it was positively correlated to P, Zn, Ca and Fe (**Figure 6**).

## 4 Discussion

### 4.1 Effects on photosynthesis and plant growth

In control plants under natural sunlight, the rate of net photosynthesis of potato leaves was similar to those recorded in potted plants grown in greenhouse, in fall-winter cycle in the same site (Caporale et al., 2023). Photosynthesis showed stable values during the stages of vegetative growth and tuber filling in ‘Colomba’, while it increased in the last period of cultivation in ‘Libra’. This is unusual for potato, since the normal pattern usually implies increasing rates from vegetative stage to flowering and decreasing values during tuberization (Vos and Oyarzun, 1987; Paradiso et al., 2019). This pattern is due to the improving light use efficiency from young to adult plants (Kitajima et al., 2002) and the subsequent decrease for plant aging, slowing the xylem transport of water, minerals, and hormones (indirectly limiting the photosynthetic rate), as well as for nutrients mobilization from leaves to tubers (directly reducing photosynthesis) during tuber filling (Dwelle, 1985).

Under natural light, the quantum yield of PSII electron transport (ΦPSII) and the linear electron transport rate (ETR) were similar in cultivars and did not change over time, whereas the maximal PSII photochemical efficiency (F_v_/F_m_) was significantly higher in ‘Libra’ than in ‘Colomba’ at 101 DAS. The F_v_/F_m_ index is a good marker of plant wellbeing, with values around 0.83 indicating a good health status and lower values revealing unfavorable conditions for photosynthetic apparatus (Maxwell and Johnson, 2000). This highlights that ‘Colomba’ plants perceived as stressful factors some cultivation conditions in our experiment.

Net photosynthesis was unaffected by light spectrum and phenological phase in ‘Colomba’, while it was higher under R:B 1:1 light supplementation compared to control, and increased over time in both these treatments in ‘Libra’. In principle, the promoting effect on NP of the enrichment of the solar spectrum with R and B is not surprising, since these wavelengths are the most efficient in sustaining plant assimilation. However, our results confirm that the sensitivity of photosynthetic activity to light quality is genotype-dependent, as highlighted in our preceding experiment in phytotron, comparing LED light (R:B 8:1) to white light (fluorescent tubes), in which we observed a different response to light source in the cultivars ‘Avanti’ and ‘Colomba’ (Paradiso et al., 2019). Our previous data also suggested that light spectrum can have a different impact on photosynthesis depending on the plant developmental stage and the most sensitive seems to be the vegetative growth (Paradiso et al., 2019).

In both cultivars, lighting treatments did not influence photochemical parameters over time. However, leaf greenness (as SPAD index) in the vegetative phase increased under both red-blue light integrations in ‘Colomba’ while was unaffected by light spectrum in ‘Libra’, compared to CNT, and decreased during the bulking stage, as expected, in both the cultivars. ‘Colomba’ showed higher chlorophyll and carotenoid levels in the vegetative stage, indicating a robust photosynthetic capacity, implying a better adaptability to changing light environment, which could contribute to explain the lack of relevant effects of light spectrum on photosynthesis. Under RB 2:1, ‘Colomba’ achieved peak chlorophyll levels, although this treatment led to significant pigment degradation during senescence. ‘Libra’, having lower chlorophyll content during the vegetative stage compared to ‘Colomba’, showed a better pigment retention during senescence, particularly in CNT, revealing to maintain photosynthetic pigments longer under less stressful conditions.

Under natural light, the biomass accumulation in both the aerial and hypogeous parts was significantly higher in plants ‘Libra’ and also tuber yield was greater (+26.6%) than in ‘Colomba’, as expected on the basis of technical sheets of the cultivars provided by the breeder (www.hzpc.com).

The potato cultivars showed different responses to light spectrum in the growth of both the aerial and hypogeous parts, hence in tuber production. In ‘Colomba’, RB 2:1 LED light improved the yield per plant by increasing the number of tubers, without affecting the leaf and root development, compared to CNT. This result is presumably due to greater proportion of R light, which is more efficient than B in sustaining carbon assimilation, allowing a greater translocation of assimilates from leaves to tubers. Accordingly, in plants grown in growth chamber under four LED spectra, white (W), RW 2:1, BW 2:1, and RBW 1:1:1 (total light intensity 300 μmol m^−2^ s^−1^, 11/13 h light/dark), RW determined the highest leaf chlorophyll content and rate of tuber bulking in the final growth period, and the highest total yield (due to higher mean tuber weight) (He et al., 2021). Similarly, when comparing W, R, B, alone and RB 1:1 light, R light delayed the leaf senescence, prolonging tuber bulking and increasing the proportion of larger tubers, finally giving the highest yield. In accordance, red light has been recognized to promote biomass accumulation in storage organs of radish (*Raphanus sativus* L.) (Drozdova et al., 2001). Conversely, B light can accelerate the carbohydrate metabolism, hastening tuberization through faster sucrose transport and tuberization signal transmission from leaves to tubers, giving a yield higher than white but lower than R light (He et al., 2020). Consistently, several studies showed that B prompted biomass accumulation in storage organs (Yorio et al., 2001; Samuolienė et al., 2011). Differently from ‘Colomba’, the growth of plants ‘Libra’ was negatively influenced by RB 2:1 light, at both the aerial and hypogeous levels. This confirms interactions between light spectrum and genotype, as already shown in potato ‘Avanti’ and ‘Colomba’ grown in phytotron under fluorescent white light and R:B 8:1 LEDs, in which we found that RB increased photosynthesis and tuber yield in both the cultivars, but influenced differently the leaf development (Paradiso et al., 2019). In general, it is conceivable that tuber bulking begins when the aerial plant part has reached a critical size to sustain tuber development and this, in turn, influences the tuberization earliness.

### 4.2 Metabolic and mineral profile of leaves

In natural light conditions, potato cultivars ‘Colomba’ and ‘Libra’ showed significant differences in the leaf accumulation of amino acids, starch, and sugars. In leaves, ‘Colomba’ plants exhibited higher levels of most amino acids, particularly essential AA including BCAAs, and proteins, while ‘Libra’ had higher glucose contents. The higher constitutive concentration of amino acids in ‘Colomba’ plants and their compact size, characterized also by smaller leaves, suggests a halophytic-like behaviour. This is confirmed by the attitude of ‘Colomba’ plants to thrive also in salty or drought environments (Caporale et al., 2023). Usually, plants would use Na^+^ as a cost-effective osmolyte to lower water potential and uptake water and nutrients. In the absence of elevated Na^+^ concentrations, they synthesize free amino acids but at the expense of growth. The synthesis of compatible metabolites has a cost of 50–70 mol of ATP per mole (Raven, 1985) and occurs through the diversion of intermediate metabolites (Parida and Das, 2005). This mechanism delays plant growth but, decreasing water potential, ensures the uptake of water and nutrients under salinity or water deficit, while the compatible osmolytes also protect and stabilize membranes and proteins (Ciriello et al., 2024). The metabolic strategy of ‘Colomba,’ with its constitutive adaptation to salinity and water-deficit stress, highlights its potential use in marginal or degraded soils where irrigation water may brackish/saline. This feature makes it particularly valuable in the context of climate change, where increasing salinity and drought pose threats to global agriculture. In contrast, ‘Libra,’ with its higher glucose content and greater reliance on alternative metabolic pathways, may perform better under more mild climatic conditions, focusing on yield optimization rather than stress adaptation. This metabolic differentiation suggests that these cultivars can complement each other depending on environmental conditions, allowing tailored cultivation strategies. The RB 1:1 light treatment, with an equal ratio of red to blue light, enhanced amino acid accumulation and carbohydrate metabolism, leading to improved growth and yield, especially in ‘Colomba’. This treatment also induced higher polyphenol content suggesting a stronger stress response. The ability of ‘Colomba’ to respond positively to RB 1:1 treatment enhancing stress tolerance, further emphasizes its resilience and possible use in controlled-environment agriculture, where light spectra can be precisely manipulated to increase stress responses and yield. This adaptability under precise lighting conditions makes ‘Colomba’ a candidate for innovative growth systems, including vertical farming and extraterrestrial agriculture. Plants progress from 101 DAS to 129 DAS caused a significant reshaping of metabolites profile, in particular GABA, methionine, starch, BCAAs, and phenylalanine, which increase, while proteins, ornithine, polyphenols, chlorophyll a, and glutamate, which decrease. The increase in GABA levels can help supporting the leaves while they are exporting nutrients. In fact, GABA has a strong antioxidant effect against ROS, helping to manage stress, maintain the stability of pH, and protect photosynthesis, while still supporting the Krebs cycle by GABA shunt and metabolic balance under increased nutrient export demands (Carillo, 2018)​. Also, methionine increased in senescing/nutrient-exporting potato leaves probably because it is a precursor for ethylene, a hormone that regulates leaf senescence and facilitates nutrient remobilization to tubers. Additionally, methionine is involved in the synthesis of polyamines, which support leaf tissue survival under aging-related export to tubers. In fact, polyamines can delay the onset of leaf senescence by stabilizing membranes and protecting cellular structures from oxidative damage thanks to their high free radicals scavenging activity, thereby maintaining cellular integrity during the nutrient export process​ (Cai et al., 2015). The accumulation of starch, while indicating still active photosynthesis in the aged leaves, also reflects a shift away from using carbon skeletons for amino acid synthesis, particularly for structural proteins (Carillo et al., 2011). This finding highlights a change in metabolic priorities to support the energy-intensive process of nutrient remobilization to the developing tubers. The accumulation of branched-chain amino acids (BCAAs, leucine, isoleucine, and valine) in senescing potato leaves compared to vegetative ones is linked to their anaplerotic and antioxidant properties. BCAAs play a key role as anaplerotic substrates for the tricarboxylic acid (TCA) cycle, supplying intermediates to the TCA cycle, essential for energy production and metabolic balance during stress-related ageing and nutrient export to tubers​ (Woodrow et al., 2017). Moreover, BCAAs help in mitigating oxidative stress by acting as free radical scavengers and supporting the synthesis of glutathione, a major antioxidant in plant cells (Ingrisano et al., 2023). ​ During the nutrient export phase in potato leaves, also phenylalanine increased. It may support the enhanced synthesis of secondary metabolites crucial for structural integrity and defence of leaf ageing. Among the metabolites that can be synthesised from phenylalanine, lignin may strengthen cell walls against pathogen invasion, and flavonoids and phenolic compounds may act as antioxidants and antimicrobial agents (Jha and Mohamed, 2022). The upregulation of stress-protective metabolites such as GABA, BCAAs, and phenylalanine in aging leaves of both cultivars indicates a metabolic modulation to balance nutrient export with oxidative stress defense. In ‘Colomba,’ this adaptation appears to be more pronounced, consistent with its stress-tolerant phenotype, suggesting that this cultivar is better adapted for stressing environments requiring long-term resource remobilization.

On the contrary, protein levels, as well as amino acids (e.g., ornithine and glutamate), chlorophylls, and polyphenols, decreased as carbon and nitrogen resources are reallocated for new tubers’ support (Aluko et al., 2023)​. Clearly, glutamate can be used both for the synthesis of amides, high nitrogen-to-carbon ratio amino acids, which are efficiently transported to the growing tubers, and as a precursor for GABA and proline, helping the plant respond to oxidative stress associated with ageing and nutrient export​ (Carillo, 2018). This degradation and reallocation of molecules support the hypothesis that leaves prioritise energy storage and stress management, optimizing conditions for protective leaves from oxidative stress while promoting tuber growth and development.

The efficient distribution of nutrients to the tubers not only promotes plant development but also increases the nutritional value of the harvested potatoes. This is especially important in controlled environments such as space shuttles, where the intake of mineral nutrients is crucial to keep the space crew healthy, not only to meet the nutritional needs of astronauts, but also to compensate for the negative effects of the space environment on the human body (Smith et al., 2013). For instance, the K (abundantly accumulated in our potato tubers), once ingested by humans, helps maintaining normal levels of fluid inside our cells and it aids muscles to contract and supports normal blood pressure (Udensi and Tchounwou, 2017). The good content of minerals in the tubers of our two cultivars (‘Colomba’ > ‘Libra’) is analogous to that of tubers of other 14 cultivars, grown by Zhou et al. (2019) in Chinese soils, demonstrating as our plant growth medium, aided by fertigation, provided the plants of a good amount of easily-available nutrients. Although nutrient uptake and utilization in horticultural crop are generally affected by light quality, intensity, and photoperiod (as reviewed by Xu et al., 2021), we found that the effect of supplemental LED lighting with RB 1:1 and RB 2:1 was statistically significant only for Zn (RB 2:1>RB 1:1) and Cu (RB 1:1>RB 2:1) concentrations in potato tubers. This may be due to mobile signalling molecules from shoot to root (whose presence could be affected by light quality), triggering the expression of nutrient use-related genes and regulating the root morphogenesis to foster the nutrient uptake (Xu et al., 2021). A non-significant effect of light quality on the ionomic profile of potato tubers was also recognized by Paradiso et al. (2019) growing two cultivars (‘Colomba’ and ‘Avanti’) in a phytotron experiment. Nevertheless, considering the significant increase of potato dry biomass induced by supplemental LED lighting, we can assume that the nutrient content (which can be calculated multiplying the nutrient concentrations by dried tuber biomass) was likely ameliorated by LED red-blue light treatments (RB 1:1 and RB 2:1). In other words, even if the light treatments had a minimal effect on mineral concentrations in the potato tubers, it conceivably had a greater (likely significant) impact on the amounts of nutrients accumulated in the potato dry matter of LED-treated plants.

### 4.3 Cultivar and light effects on tuber quality

‘Colomba’ tubers showed higher concentrations of total and essential amino acids, such as arginine, histidine, lysine, threonine, methionine, and phenylalanine, along with primary amino acids like proline, tyrosine, serine, glycine, and asparagine. This confirms that this cultivar has a constitutive metabolic profile geared towards amino acid synthesis and accumulation, which could be linked to better stress tolerance and adaptability to varying growth conditions. In fact, these metabolites act as compatible solutes and antioxidants, helping the plant manage osmotic balance and oxidative stress. In contrast, ‘Libra’, showed higher starch content and dry weight, larger size and greater length, advantageous for tuber quality and yield. This indicates that ‘Libra’ follows a different metabolic strategy, prioritizing energy storage and tuber growth rather than metabolic diversion. ‘Libra’’s higher starch content, larger tubers, and greater length reflect its focus on energy storage, which enhances its yield potential under stable growth conditions. This cultivar having greater leaf area and aerial biomass, focuses its strategy on maximizing photosynthetic capacity and growth. However, the high levels of GABA in ‘Libra’ suggest that it is anyway able to cope with osmotic and oxidative stress. In fact, high GABA levels in the growing tuber aid in buffering cytosolic acidosis by consuming protons during its synthesis. As a zwitterion, GABA functions as an osmolyte without causing toxicity while maintaining water potential balance during cellular dehydration. Additionally, GABA has a significant ROS scavenging activity, protecting membrane and macromolecule structures and activity. GABA can also have positive effects on human health. It acts as a hypotensive agent and enhances the immune system under stress, and it may contribute to the prevention of cancer and diabetes, as well as help control blood cholesterol levels (Carillo, 2018).

In ‘Libra’ RB 2:1 treatment affected photosynthesis and overall plant growth as expected (Izzo et al., 2020), but further amplified the plant capacity to accumulate higher levels of starch, producing a fewer number of tubers with bigger size and weight, again indicating a focus on carbohydrate storage and growth. Moreover, ‘Libra’’s tubers under RB 1:1 and RB 2:1 treatments showed higher percentage of DW relative to fresh weight, indicating efficient water management and nutrient storage. ‘Colomba’ cultivar shows a pronounced response to RB 2:1 treatment, with increased tuber number and hypogeous biomass, highlighting its adaptability and capacity to enhance tuber production under stress conditions. Jungandreas et al. (2014) showed that a shift from blue light to red light led to carbohydrate accumulation, while the switch from red to blue light caused carbon synthesis to shift to protein synthesis. Chen et al. (2020) also found that potato plantlets accumulated more carbohydrates under red light than under blue light. On the contrary, our data showed that while both light ratios can enhance tuber yield, the 2:1 red to blue light ratio has a more pronounced effect on improving yield for both potato varieties, ‘Colomba’ and ‘Libra’, but the treatments did not affect starch or protein contents of tubers.

## 5 Conclusions

Under natural light conditions, plant growth and tuber yield in plants grown in pot in greenhouse in winter-spring period were greater in ‘Libra’ then in ‘Colomba’. Plants revealed a genotype-dependent response to light spectrum in terms of both agronomical and metabolic traits. Indeed, in ‘Colomba’ the tuber production increased while in ‘Libra’ decreased under RB 2:1 light integration. ‘Colomba’ plants constitutively prioritises accumulating free amino acids and maintained a compact size, able to endure and adapt to stressful environments. Besides, ‘Colomba’ accumulated higher levels of free amino acids and polyphenol levels, which enhanced plant antioxidant properties and stress response, but also tuber nutraceutical quality. Differently, ‘Libra’ maximizes yield through enhanced carbohydrate synthesis and export, favouring growth and productivity under favourable conditions, and also showed high levels of GABA that boost its premium tuber quality.

These insights underscore the importance of choosing the proper plant genotype and lighting strategy based on the specific environmental conditions and desired outcomes. Our findings could be useful in indoor cultivation (i.e., vertical farming) as well as in space research on potato, as this crop is a candidate for plant-based regenerative support systems for long-term missions in Space.

## Data Availability

The original contributions presented in the study are included in the article/**Supplementary Material**. Further inquiries can be directed to the corresponding author.

## Supplementary material

Supplementary Table 1 Significance of statistical differences in Physiological parameters in potato plants cv. ‘Colomba’ and ‘Libra’ grown under natural light (control, CNT) and natural light shaded at 30% and integrated with red and blue LED light at 1:1 (RB 1:1) and 2:1 (RB 2:1) ratios. Mean values ± Standard Errors; n=3. Different letters indicate significant differences according to Tukey’s multiple-range test (p<0.05). ns: not significant; * p<0.05; ** p<0.01; *** p<0.001.
