# Supplementary material for: Enhancing tuber yield and nutraceutical quality of potato by supplementing sunlight with LED red-blue light
Source: Front Plant Sci. 2025 Mar 10;16:1517074. doi: 10.3389/fpls.2025.1517074 (PMC11931653; doi:10.3389/fpls.2025.1517074)
Supplement: Supplementary Table 1 — Significance of statistical differences in Physiological parameters in potato plants cv. ‘Colomba’ and ‘Libra’ grown under natural light (control, CNT) and natural light shaded at 30% and integrated with red and blue LED light at 1:1 (RB 1:1) and 2:1 (RB 2:1) ratios. Mean values ± Standard Errors; n=3. Different letters indicate significant differences according to Tukey’s multiple-range test (p<0.05). ns: not significant; * p<0.05; ** p<0.01; *** p<0.001. [file DataSheet1.pdf]

Table S1 - Significance of statistical differences in Physiological parameters in potato plants cv. 'Colomba' and 'Libra' grown under natural light (control, CNT) and natural light shaded at 30% and integrated with red and blue LED light at 1:1 (RB 1:1) and 2:1 (RB 2:1) ratios. Mean values ± Standard Errors; n=3. Different letters indicate significant differences according to Tukey's multiple-range test ($p<0.05$). ns: not significant; * $p<0.05$; ** $p<0.01$; *** $p<0.001$.

	Cultivar	DAS	Cultivar x DAS
NP	***	ns	***
gs	***	***	ns
E	***	***	ns
PSII	**	***	ns
ETR	ns	ns	ns
Fv/Fm	***	ns	ns
SPAD	***	***	ns
